# Group IB Metal‐Based Nanomaterials for Antibacterial Applications

**DOI:** 10.1002/smsc.202400412

**Published:** 2025-03-09

**Authors:** Xuezhi Zhao, Hongyu Wang, Yun Sun, Jin Zhang, Huiyu Liu

**Affiliations:** ^1^ Beijing Advanced Innovation Center for Soft Matter Science and Engineering State Key Laboratory of Organic‐Inorganic Composites Bionanomaterials & Translational Engineering Laboratory Beijing Key Laboratory of Bioprocess Beijing Laboratory of Biomedical Materials Beijing University of Chemical Technology Beijing 100029 China

**Keywords:** antibacterial mechanisms, antibacterial nanomaterials, bacterial infections, group IB metal‐based nanomaterials, group IB metals

## Abstract

Pathogenic bacteria pose significant threats to human health. In recent years, escalating bacterial resistance against antibiotics has diminished their efficacy in treating infections like pneumonia, tuberculosis, and sepsis, making some cases virtually untreatable. Hence, there is an urgent demand for novel approaches to combat bacterial threats. Group IB metal‐based nanomaterials including copper, silver, and gold have attracted considerable attention in the field of antibacterial research owing to their remarkable broad‐spectrum bactericidal properties. Their high efficacy, ease of synthesis, and amenability for functionalization render group IB metal‐based nanomaterials highly promising for diverse applications in the antibacterial domain. This review comprehensively elucidates on the bactericidal mechanisms and applications of IB‐group metal‐based nanomaterials in addressing bacterial infections. Additionally, insights into challenges associated with utilizing group IB metal‐based nanomaterials for such purposes while outlining future directions of research are provided.

## Graphical abstract

Group IB metal‐based nanomaterials exhibit excellent antibacterial properties, which can kill bacteria through metal ion toxicity, reactive oxygen species, and photothermal effects. The current research on these nanomaterials mainly focuses on two directions: exploring how to achieve stronger and more comprehensive antibacterial performance and investigating how to use smaller dose and achieve more precise release for antibacterial therapy.

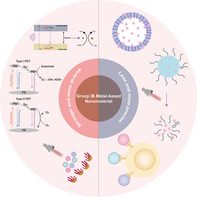

## 1 Introduction

**Figure 1:**
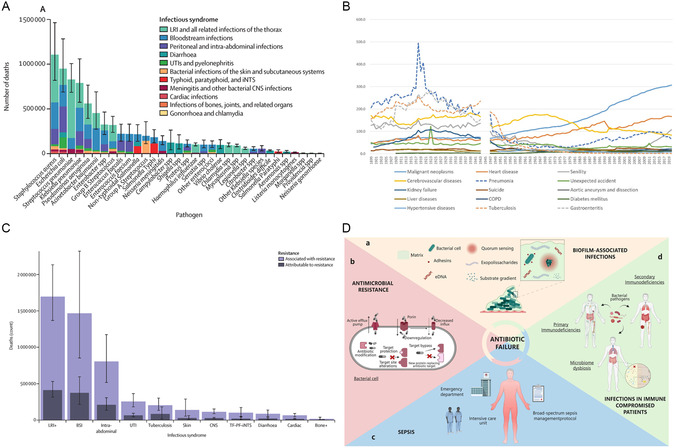
A) Global number of deaths caused by pathogens and related infectious syndromes in 2019. Reproduced with permission.^[^ ^6^ ^]^ Copyright 2022, Elsevier Ltd. B) Annual transition of the mortality rate in Japan from 1899 to 2020. (The ordinate is mortality per 100 000, the horizontal coordinate is the year). Reproduced with permission.^[^ ^9^ ^]^ Copyright 2022, MDPI AG. C) Global deaths attributable to and associated with bacterial antimicrobial resistance in 2019. Reproduced with permission.^[^ ^23^ ^]^ Copyright 2022, Elsevier Ltd. D) Common causes of antibiotic treatment failure. Reproduced with permission.^[^ ^25^ ^]^ Copyright 2023, Churchill Livingstone.

Pathogenic bacteria can induce infections and diseases in various parts of the human body, posing a great threat to human health.^[^ ^1^, ^2^ ^]^ Since the word “bacterium” was coined in 1828, humans have officially begun the fight against pathogenic bacteria.^[^ ^3^ ^]^ Countless lives have been taken away during multiple large‐scale outbreaks of bacterial infections in history,^[^ ^4^ ^]^ such as typhoid caused by *Salmonella typhi*; the Black Death caused by *Yersinia pestis*; cholera caused by *Vibrio cholerae*. According to statistics, three people die from tuberculosis caused by *Mycobacterium tuberculosis* every minute.^[^ ^5^ ^]^ In 2019, it was estimated that 7.7 million deaths were related to 33 bacterial pathogens globally, accounting for more than one‐eighth of total deaths worldwide that year (**Figure** **1**A).^[^ ^6^ ^]^ Nowadays, “ESKAPE” pathogens including *Enterococcus faecium* (*E. faecium*), *Staphylococcus aureus* (*S. aureus*), *Klebsiella pneumoniae*, *Acinetobacter baumannii* (*A. baumannii*), *Pseudomonas aeruginosa* (*P. aeruginosa*), and *Enterobacter* species are still posing a huge threat to human lives.^[^ ^7^ ^]^

Since the discovery of penicillin in 1928, antibiotic treatment has been widely recognized as the primary approach for bacterial infections control.^[^ ^8^ ^]^ The introduction of antibiotics led to a significant decline in deaths from infectious diseases such as tuberculosis and gastroenteritis after World War II (Figure 1B).^[^ ^9^ ^]^ An increasing variety of antibiotics has become available as antibiotic research deepens, including β‐lactam, aminoglycoside, quinolone, tetracycline antibiotics, etc.^[^ ^10^, ^11^, ^12^, ^13^, ^14^, ^15^ ^]^ Antibiotics typically bind to bacteria targets, such as cell membranes, extracellular enzymes, or RNA components of bacterial ribosomes, to deactivate them and disrupt the growth and reproduction processes of bacteria.^[^ ^16^, ^17^, ^18^, ^19^ ^]^ However, bacteria often employ spontaneous mutations during chromosome replication to modify their antibiotic targets, thereby developing antimicrobial resistance (AMR).^[^ ^20^ ^]^ Due to the emergence of AMR, infections become increasingly challenging or even untreatable, consequently increasing the risk of disease spread, severe illness, disability, and death.^[^ ^21^ ^]^ Therefore, AMR has been listed as one of the top global health threats by the World Health Organization,^[^ ^22^ ^]^ which is directly responsible for 1.27 million deaths and associated with 4.95 million deaths worldwide in 2019 (Figure 1C).^[^ ^23^ ^]^ However, the current pace of antibiotic development lags behind the emergence of bacterial resistance, even multidrug resistance, which may become lethal in the future if effective measures are not taken.^[^ ^24^ ^]^ Moreover, biofilm‐related infections, infections in immunocompromised patients, and sepsis are also common causes of antibiotic treatment failure (Figure 1D).^[^ ^25^ ^]^

Prophylactic vaccines are used to prevent our body from bacterial infection and have shown unique advantages in the field of antibacterial.^[^ ^26^ ^]^ Prophylactic vaccines typically contain multiple immunogenic epitopes, requiring pathogens to undergo more mutations to develop resistance against them.^[^ ^27^, ^28^ ^]^ In addition, in contrast to antibiotic treatments, which are administered post‐infection with billions of bacteria present, vaccines are employed preemptively when the body is exposed to a relatively small number of pathogens (hundreds or thousands), thereby significantly reducing the likelihood of bacterial mutation.^[^ ^29^ ^]^ The use of vaccines can effectively reduce the reliance on antibiotics, thereby reducing the emergence of antibiotic resistance.^[^ ^30^, ^31^ ^]^ Unfortunately, the majority of vaccines developed against prominent drug‐resistant pathogens are still undergoing preclinical and clinical evaluation and there is a lack of vaccine candidates for certain common pathogenic strains, such as *P. aeruginosa*, *Helicobacter pylori*, *Campylobacter jejuni*, *Enterobacter spp*, *E. faecium*, and *A. baumannii.* ^[^ ^32^ ^]^ In addition, the development of a new vaccine is a lengthy process, usually 10 to 20 years; thus, relying solely on vaccines for prevention is limited.^[^ ^28^ ^]^ Therefore, the development of rational new strategies to combat bacterial infections is indispensable.

With the advancement of nanotechnology, nanomaterials (NMs) with distinctive physicochemical properties have emerged as novel prospects in the field of antibacterial applications.^[^ ^33^ ^]^ In comparison to conventional antibiotics, antibacterial NMs possess enhanced membrane permeability, multiple antibacterial mechanisms, and augmented antibacterial efficacy.^[^ ^34^ ^]^ Additionally, antibacterial NMs leverage their antibacterial effects by inherent toxicity of ions release, generating photothermal properties or reactive oxygen species (ROS), and thereby can circumvent resistance‐related concerns.^[^ ^35^ ^]^ Many metal‐based NMs, including zinc (Zn), iron (Fe), magnesium (Mg), titanium (Ti), silver (Ag), and copper (Cu), have demonstrated significant antibacterial properties. For example, Zn^2+^ ions can interact with cellular components such as proteins, lipids, and nucleic acids, thereby disrupting bacterial metabolic processes, cell division, and replication, ultimately leading to bacterial death.^[^ ^36^, ^37^ ^]^ Metal oxides such as ZnO, MgO, and TiO_2_ nanoparticles (NPs) can generate ROS under external stimuli, which contribute to their antibacterial effects.^[^ ^38^, ^39^, ^40^, ^41^ ^]^ Iron oxide NPs and Al_2_O_3_ exhibit antibacterial activities by producing ROS through enzyme‐like catalytic actions.^[^ ^42^, ^43^, ^44^, ^45^ ^]^

As a matter of fact, metal‐based antibacterial therapies were described long before the development of modern science. For example, Ag has been used since ancient times to store drinking water and treat infections such as wounds and ulcers.^[^ ^46^ ^]^ Silver nitrate eye drops have been introduced in Germany to prevent neonatal gonococcal ophthalmitis since 1881.^[^ ^47^ ^]^ In recent decades, numerous metal‐based antibacterial agents with well‐characterized properties have been extensively studied. Among these, Group IB metals, including Cu, Ag, and gold (Au), has experienced rapid growth in their application in antibacterial treatments. Due to their metal ion toxicity and catalytic properties, these metals exhibit broad‐spectrum antibacterial effect against both Gram‐positive and Gram‐negative bacteria.^[^ ^48^ ^]^ Colloidal nanosilver was officially registered as a bactericidal material in 1954.^[^ ^49^ ^]^ Cu was recognized as an effective metal antimicrobial by the United States Environmental Protection Agency (EPA) in 2008.^[^ ^50^, ^51^ ^]^ The application of Au in the antibacterial field started much later. The research advancements in Group IB metal‐based NMs offer substantial guidance for the development of other metal‐based antibacterial NMs and their antibacterial mechanisms are particularly representative. Therefore, this review focuses on the research progress of Group IB metal‐based NMs for antibacterial applications, providing a comprehensive discussion on the action mechanisms of Cu, Ag, and Au‐based NMs from a materials science perspective. Additionally, we address the current challenges and the potential future research directions for group IB metal‐based NMs in antibacterial treatment.

## 2 Antibacterial Mechanisms of Group IB Metal‐Based Nanomaterials

As members of the same group, Cu, Ag, and Au have similar outer electron configurations, thus exhibiting alike physicochemical properties. Group IB element‐based NMs can effectively inhibit and eradicate bacteria through ionic toxicity, generation of ROS, and photothermal effects. **Table** **1** provides a summary of research on the application of Group IB metal‐based NMs in the antibacterial field that has been reported over the past 5 years.

**Table 1:** Evaluation of antibacterial activity of group IB metal NMs.

NM composition	Size and shape	Tested microorganism	Minimum inhibitory concentration (MIC)	Antibacterial mechanism	Application	References
Nanosilver	Spherical shape ≈55.20 nm	*S. aureus*	12.5 μg mL^−1^	Not mentioned	Coatings of medical equipment, water filters, paints, detergents, respirators, and many other consumer products	[62]
Polycationic silver (pAg) NCs	<3 nm	*S. epidermidis*, *Escherichia coli* (*E. coli)*, *F. nucleatum*, *S. aureus*, *P. aeruginosa*, *S. sanguinis*	3.375, 3.375, 3.375, 6.75, 6.75, 3.375 μg mL^−1^	Released silver ion	Not mentioned	[131]
Silver nanocrystal clusters	1.6 ± 0.3 nm	*E. coli*, *S. aureus,*	5, 10 μg mL^−1^	Released silver ion	Not mentioned	[132]
Ag_2_O_2_ NPs	Spherical shape, 43 ± 10 nm	*E. coli*, *P. aeruginosa*, *S. aureus*, and MRSA	14.0 ± 0.2, 33.1 ± 1.0, 26.0 ± 0.3 34.2 ± 1.9 μg mL^−1^	US‐assisted ROS production, photothermal therapy (PTT)	Against wound infection	[144]
Doxycycline hyclate(DX)‐Ag–AgCl NPs	Spherical shape, 5–20 nm	*S. aureus*, *E. coli*	8, 8 μg mL^−1^	Not mentioned	Not mentioned	[146]
Ag NPs‐incorporated nanoporous carbon nitride	4–4.8, 5–8 nm	*E. coli*	32, 16 μg mL^−1^	Not mentioned	Medical devices, food packaging, disinfectant, and wound dressing	[195]
Modified graphene/Ag	70 nm	*E. coli*, *S. aureus*	0.6 mg mL^−1^	Released silver ion	Not mentioned	[205]
Ag/COF_TGTp_ (covalent MOF)	950 nm	*E. coli*, *S. aureus*	100, 50 μg mL^−1^	Released silver ion	Wound dressing	[206]
PolyCu‐MOF embedded with Ag NPs	600 nm	*E. coli*, *S. aureus*	10 μg mL^−1^	ROS generation, released silver ion	Against wound infection	[244]
CuO NPs, Cu_2_O NPs	≈30, ≈40 nm	*E. coli*	0.1, 0.05 mM	ROS generation, released copper ion	Not mentioned	[160]
Ag@Cu_2_O	Spherical shape, 120 nm	*S. aureus*, *P. aeruginosa*	56.8 μg mL^−1^	PDT and released ion	Novel antifouling agents for marine antifouling	[180]
Cu_2_O/Ag	MS/triangular sheets	*E. coli*, *S. aureus*	7.8 and 15.6, 3.9 and 7.8, 0.5 and 1.0, 2.0 and 3.9 μg mL^−1^	ROS generation, released copper ion and mechanical damage	Not mentioned	[183]
Pomegranate‐Like CuO_2_@SiO_2_ Nanospheres	Spherical shape, 35 ± 42 nm	*E. coli*, *S. aureus*	56 ± 3 and 49 ± 8 μg mL^−1^ (pH 5.0), 75 ± 6 and 65 ± 9 μg mL^−1^ (pH 7.4)	ROS generation	Against wound infection	[197]
Graphdiyne nanowalls wrapped hollow copper sulfide	Nano cube	*E. coli*, *S. aureus*	17 ± 1, 14 ± 1 μg mL^−1^	ROS generation, PTT	Against wound infection	[200]
Copper(I) Oxide Incorporated in Zeolite	50–100 nm	*E. coli*, *S. aureus*	25 μg mL^−1^	ROS generation, released copper ion and mechanical damage	Not mentioned	[208]
Tannic acid (TA) capped copper NCs (Cu NCs)	1.0 ± 0.7 nm	*S. aureus*, *B. subtilis*	5, 15 μg mL^−1^	Released copper ion	Not mentioned	[222]
BSA‐encapsulated copper sulfide (CuS) nanocrystals	Leaf‐like structure, 500 nm	*S. aureus*, *S. enteritidis*, *E. coli*, *P. aeruginosa*	24–35 μg mL^−1^	ROS generation, PTT and PDT	Against wound infection	[225]
Cu NCs	<2 nm	MRSA	0.1 mM	PDT	Against wound infection	[233]
Au/Fe–Ag_2_O_2_ NPs	Spherical shape, 70 nm	*E. coli*, *P. aeruginosa*, *S. aureus*, MRSA, *E. faecalis*	More than twice as small as penicillin, levofloxacin, gentamicin	ROS generation	Against catheter‐associated urinary tract infection	[182]
AuNPs modified with amine‐ or thiol‐tethered phenylboronic acids	4 ± 0.8 nm	*E. coli*, *S. aureus*	3–9 μg mL^−1^	PDT	Intra‐abdominal infection	[236]
D‐maltose‐capped gold NCs (AuNC‐Mal)/thiourea (TU)	≈1.8 nm	*P. aeruginosa*, *E. coli*, *K. pneumoniae*, *S. aureus*, *M. smegmatis s*	1, 4, 1, 0.5, 0.5 μg mL^−1^	Released gold ion	Not mentioned	[237]
Chitosan‐Based Au NPs	Spherical shape, 20–120 nm	*B. subtilis*, *S. aureus*, *P. aeruginosa*, *K. Oxytoca*	6.25, 6.25, 1.56, 3.12 μg mL^−1^	Synergistic effects of AuNPs and Chi	Bandage	[238]
Ag NPs	Cubes, discs, pseudospheres, truncated triangular	*E. coli*	Not mentioned	Released silver ion, mechanical damage	Not mentioned	[127]
Ag NPs	Sphere shape and quasi‐spherical shape, ≈75 nm	*E. coli*	Not mentioned	Released silver ion	Not mentioned	[129]
AgNPs with different organic ligand	Spherical shape, ≈10 nm	*E. coli*	Not mentioned	Released silver ion	Not mentioned	[52]
Ag NPs	Sphere shape, disk shape and triangular plate shape	*E. coli*, *S. aureus*, *P. aeruginosa*	Not mentioned	Released silver ion	Not mentioned	[124]
Silver NW film (Ag NW)	54 ± 6 nm	*E.coli, S. aureus*	Not mentioned	Released silver ion	Antibacterial coating	[134]
The B subunit of the subtilase cytotoxin (SubB) ‐modified silver NPL	Triangular shape, 82.5 ± 1.0, 14.7 ± 0.77 nm	*Salmonella typhimurium*	Not mentioned	Released silver ion, mechanical damage	Severe infectious diseases caused by intracellular bacteria	[137]
PDA@Ag NSs	2D NS	*E. coli*, *S. aureus*	Not mentioned	Released silver ion, mechanical damage	Food packaging	[138]
Ag_2_O NPs	Spherical shape, 80–90 nm	*E. coli*, *S. aureus*	Not mentioned	ROS generation	Not mentioned	[139]
AgO NPs	10 nm	*E. coli*, *S. aureus*	Not mentioned	Released silver ion	Not mentioned	[140]
Ag_2_O NPs	1.5 μm	*S. aureus*, *P. aeruginosa*, MRSA	Not mentioned	Released silver ion	Transparent antimicrobial coatings	[141]
Ag_2_O NPs	20 nm	*S. aureus*	Not mentioned	Not mentioned	Wound dressing	[142]
Mixed phase Ag_2_O NPs	49.76 nm	*E. coli*, *P. aeruginosa*, *S. aureus*, *B. subtilis*	Not mentioned	Released silver ion	Not mentioned	[143]
Ag_2_S NCs	Not mentioned	*E. coli*, *P. aeruginosa*	Not mentioned	ROS generation	Against wound infection	[145]
BU‐TiO_2−*X*_/Ag_3_PO_4_	Urchin‐like structure	*E. coli*, *S. aureus*	Not mentioned	PDT, released silver ion and mechanical damage	Not mentioned	[176]
Ag/Bi_2_MoO_6_ NPs	116 nm	MRSA	Not mentioned	ROS generation, released silver ion and mechanical damage	Against wound infection	[186]
Ag/CeO_2_ NPs	17 nm	*E. coli*, *S. aureus*	Not mentioned	PDT	Biological pollutants removal	[188]
AgNP‐CNT(carbon nanotubes) nanohybrid	Spherical, snowflake and cube shape, 95–200 nm	*S. aureus*	Not mentioned	Not mentioned	Against wound infection	[196]
Ag/AgO/g‐C_3_N_4_	Spherical shape	*E. coli*	Not mentioned	PDT, released silver ion	Not mentioned	[199]
Ag/rGO	10–100 nm	*E. coli*	Not mentioned	ROS generation, released silver ion	Water disinfection, electric and biomedical devices	[203]
Ag‐MOF@HA	Polyhedral morphology	*S. aureus*	Not mentioned	ROS generation	Intrauterine adhesion (IUA) treatment	[215]
Ag_2_S quantum dot/mSiO_2_ NPs	Spherical shape, 9.7 ± 1.8 nm	*E. coli*, MRSA	Not mentioned	ROS generation, released silver ion and PTT	Against wound infection	[218]
Tannic acid chelated‐Ag (TA‐Ag) nanozyme	5 nm	*E. coli*, *S. aureus*	Not mentioned	ROS generation	Antibacterial wound dressing, self‐adhesive biosensor, or implantable and wearable electronic appliance	[235]
UiO‐66‐NH_2_–Ag	≈60 nm	*E. coli*, *B. subtilis*	Not mentioned	ROS generation, released silver ion	Against wound infection	[243]
Ag NPs‐decorated 2D Co‐TCPP MOF	NS	*E. coli*, *S. aureus*	Not mentioned	ROS generation, released silver ion	Not mentioned	[246]
PDA@Cu NPs	220 nm	*E. coli*, *S. aureus*	Not mentioned	PTT	Against wound infection	[229]
rGO functionalized through copper ions	Single‐layer sheet, ≈10 μm	*E. coli*, *S. aureus*	Not mentioned	Synergetic therapy, released copper ion	Not mentioned	[64]
Copper surface	NW, NS, and microflower structures	*E. coli*, *B. subtilis*	Not mentioned	Released copper ion, mechanical damage	Coating	[153]
Ultrasmall Cu_30_ NCs	≈1.12 nm	*E. coli*, *S. aureus*	Not mentioned	Not mentioned	Against primary peritonitis	[154]
Copper cluster	<2 nm	*E. coli*, *P. aeruginosa*, *S. aureus*, MRSA, *S. epidermidis*	Not mentioned	ROS generation, mechanical damage	Against wound infection	[155]
CuO NPs	Spherical shape, 40–150 nm	*S. pneumoniae*, *S. epidermidis*, *E. coli*, *P. aeruginosa*	Not mentioned	ROS generation, electrostatic interaction	Not mentioned	[158]
CuO NPs/NRs	25.20–22.58 nm	*E. coli*, *P. aeruginosa*, *S. aureus*, *Bacillus cereus*	Not mentioned	Released copper ion	Not mentioned	[159]
CuO_2_ NPs	NW, 11 nm	*E. coli*, *S. aureus*, *P. aeruginosa*	Not mentioned	Released copper ion and H_2_O_2_	Against wound infection	[162]
Copper sulfide (Cu_2−*x*_S) nanozymes	NPL	*E. coli*, *S. aureus*	Not mentioned	ROS generation	Not mentioned	[166]
CuO/AgX (X = Cl, Br, or I)	10–30 μm	*E. coli*, *S. aureus*	Not mentioned	PDT, released ion	Not mentioned	[179]
CP@WS_2_ NFs	Floral MS	*E. coli*, *S. aureus*	Not mentioned	ROS generation, PTT	Against wound infection	[189]
Bimetallic oxide Cu_1.5_Mn_1.5_O_4_	Hollow sphere structure	*E. coli*, MRSA	Not mentioned	ROS generation	Against wound infection	[192]
Copper‐doped PBG nanozymes	Spherical shape, 150 nm	*E. coli*, *S. aureus*	Not mentioned	ROS generation, released copper ion	Against wound infection	[194]
Copper/carbon hybrid Nanozyme	Spherical shape, ≈100 nm	*S. aureus*, *P. aeruginosa*, *S. mutans*, *S. aureus*	Not mentioned	ROS generation, released copper ion	Against intestinal infection and wound infection	[201]
Ti_3_C_2_@CuS	NS	*E. coli*, *S. aureus*	Not mentioned	ROS generation, released copper ion and PTT	Not mentioned	[202]
rGO–Cu_2_O	<30 nm	*E. coli*, *S. aureus*	Not mentioned	ROS generation, released copper ion and mechanical damage	Not mentioned	[204]
Cu_2_O@CuO	Spherical shape, 8.50, 9.49, 7.68, 8.86, 15.18 nm	*E. coli*	Not mentioned	Released copper ion	Coatings	[211]
CeCu–MOF	NS	*S. aureus*, *B. subtilis*	Not mentioned	PDT	Against wound infection	[242]
3D hierarchical Cu‐MOF	Sharp‐edged NS	*S. aureus*, *B. subtilis*	Not mentioned	ROS generation, mechanical damage	Not mentioned	[248]
Cu single‐atom sites/N doped porous carbon (Cu SASs/NPC)	193 ± 19 nm	*E. coli*, MRSA	Not mentioned	ROS generation, PTT	Against wound infection	[250]
Ultrathin two‐dimensional Au‐Por NS	NS, <10 nm	*E. coli*, *S. aureus*	Not mentioned	PDT, ROS generation and released gold ion	Against wound infection	[65]
Plasmonic AuNRs and AuNBPs	107.59 ± 3.98, 31.31 ± 1.63, 82.24 ± 3.34, 24.90 ± 1.75 nm	*E. coli*	Not mentioned	PTT	Not mentioned	[174]
Au@BTO	146.8. 150.7, 158.8, 151.5 nm	*E. coli*, *S. aureus*	Not mentioned	Sonodynamic therapy	Against wound infection	[181]
Au/MoO_3–*x*_	108 nm	MRSA	Not mentioned	ROS generation, PTT	Against wound infection	[190]
RBC‐HNTM‐Pt@Au	Hollow cube structure	MRSA	Not mentioned	Sonodynamic therapy	Osteomyelitis	[191]
Au@mesoporous polydopamine(MPDA) NPs	180 nm	*E. coli*, *S. aureus*	Not mentioned	PTT	Sports injury healing	[230]
Mixed‐Charge Gold NPs	50 nm	*E. coli*, *S. aureus*	Not mentioned	Interplay between polyvalent electrostatic and non‐covalent interactions	Not mentioned	[240]
Ultrasmall Au NPs/2D MOFs	150 nm	*E. coli*, *S. aureus*	Not mentioned	ROS generation	Against wound infection	[245]

### 2.1 Antibacterial Effect Achieved through Metal Ions

**Figure 2:**
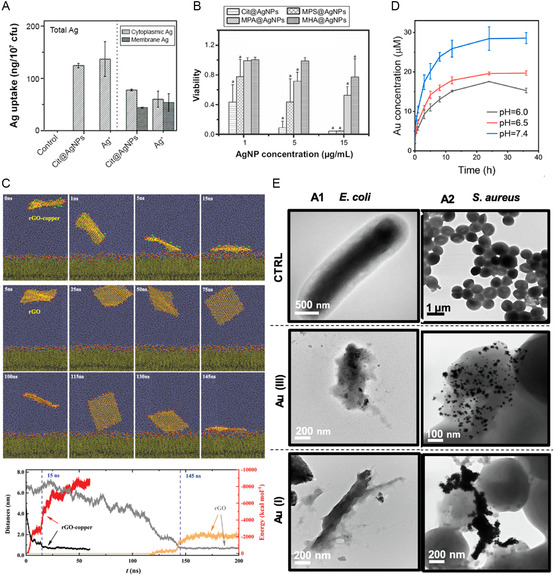
A) Total Ag uptake in *E. coli* (left) and its distribution (right). B) The viabilities of *E. coli* after 6 h exposures of Ag NPs. Reproduced with permission.^[^ ^52^ ^]^ Copyright 2017, Dove Medical Press Ltd. C) Molecular interaction of the functionalized rGO–copper composite with bacterial cell membranes. Reproduced with permission.^[^ ^64^ ^]^ Copyright 2021, Wiley‐VCH Verlag. D) Au release curves from Au‐Por in different pH buffers (6.0, 6.5, and 7.4). Reproduced with permission.^[^ ^65^ ^]^ Copyright 2023, Elsevier BV. E) transmission electron microscope micrographs of bacteria treated with ionic gold species. Reproduced with permission.^[^ ^66^ ^]^ Copyright 2023, Academic Press Inc.

One of the widely acknowledged mechanisms for metal‐based antibacterial agents involves utilizing the toxicity of metal ions to eradicate bacteria. Studies have demonstrated a positive correlation between the release of Ag^+^ and its antibacterial efficacy (**Figure** **2**A,B).^[^ ^52^ ^]^ Higher levels of released Ag^+^ from Ag NMs incorporated into other materials such as hydrogels and implants lead to enhanced antibacterial performance.^[^ ^53^, ^54^ ^]^ The release of ions by Cu NMs during the antibacterial process has also been substantiated by numerous studies.^[^ ^55^ ^]^ In contrast to Ag NMs and Cu NMs, the inherent stability of Au NMs restricts the release of Au ions, resulting in less reliance on ions release for its antibacterial activity.^[^ ^56^ ^]^

#### 2.1.1 Metal Ions Modulate the Membrane Potential of Bacterial Cell

The cell membrane is a crucial component of bacterial cells, providing protection to the bacteria while also serving as a scaffold for metabolic and regulatory proteins.^[^ ^57^ ^]^ The electric potential difference present on the bacterial cell membrane, known as membrane potential (or transmembrane voltage), plays a pivotal role in regulating various physiological activities of bacteria, holding significant importance.^[^ ^58^, ^59^ ^]^ The membrane potential of bacteria is stable at a negative value, which is conducive to the binding of positively charged metal ions through electrostatic interactions. Consequently, these positive metal ions induce changes in bacterial cell membrane potential toward neutrality and lead to alterations in membrane permeability. Moreover, the alternation on the bacterial cell membrane potential impact crucial cellular processes, including cell division and lipid‐mediated signal transduction, resulting in bactericidal effects.^[^ ^60^, ^61^, ^62^ ^]^

Ag^+^ released by Ag NMs can adhere to the cell wall and plasma membrane due to electrostatic interaction, and the adherent ions can enhance the permeability of the plasma membrane and cause damage to the membrane.^[^ ^63^ ^]^ Cu ions have also been reported to affect membrane potential. Fang et al. loaded Cu^2+^ onto reduced graphene oxide (rGO) and found that Cu^2+^ interacted strongly with negatively charged bacterial cells due to the potential difference, thereby achieving selective antibacterial effect while causing no cytotoxicity to neutral charge mammalian cells (Figure 2C).^[^ ^64^ ^]^ As for Au, a mixed‐valence Au‐porphyrin (Au‐Por) 2D coordination network was constructed by Zhao et al.^[^ ^65^ ^]^ It was observed that as the size of the Au NPs decreased, their solubility increased (Figure 2D). This allowed sustained release of Au ions that could diffuse and deposit on the surface through stable electrostatic interactions with the negative charge of the bacterial membrane, leading to perforation and lysis of the bacterial cell membrane (Figure 2E).^[^ ^66^ ^]^ Besides, Au NPs could be modified and functionalized at their surfaces to carry surface‐bound ions to achieve antibacterial effects.^[^ ^67^ ^]^

#### 2.1.2 Metal Ions Impact the Function of Bacterial Biomolecules

**Figure 3:**
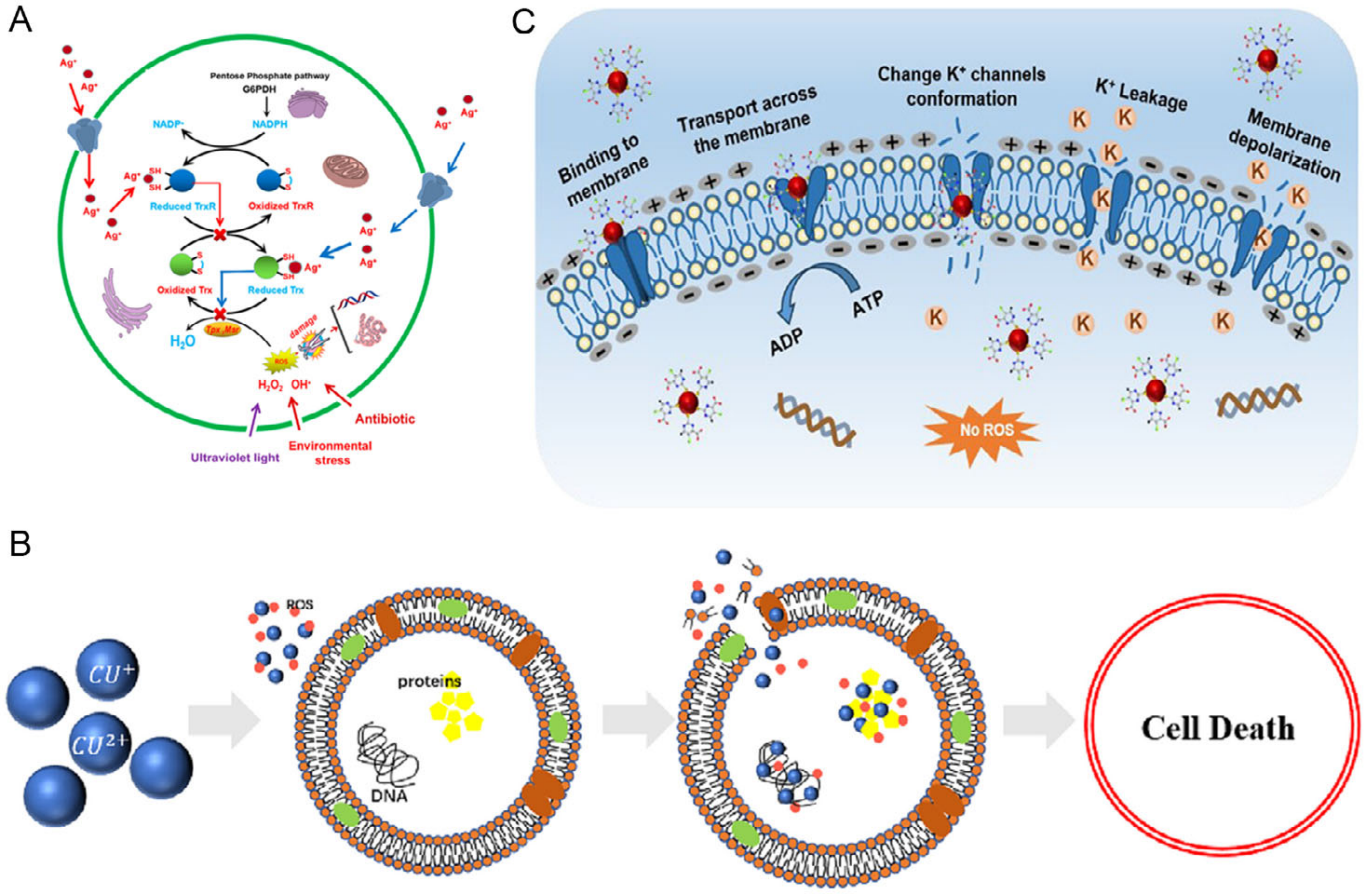
A) Proposed mechanism for Ag^+^‐induced inhibition of the thioredoxin–thioredoxin reductase system. Reproduced with permission.^[^ ^70^ ^]^ Copyright 2017, American Chemical Society. B) Antibacterial action of copper ions. Reproduced with permission.^[^ ^73^ ^]^ Copyright 2024, John Wiley and Sons Ltd. C) Illustration of the antibacterial action of anionic Au NPs. Reproduced with permission.^[^ ^77^ ^]^ Copyright 2024, American Chemical Society.

Biomolecules such as proteins and DNA play an extremely important role in various cellular activities of bacteria.^[^ ^68^, ^69^ ^]^ The Ag^+^ and Cu^2+^ released by group IB element‐based NMs can impact the function of these biomolecules in bacteria through multiple pathways to exert bactericidal effects. Sun et al. reported that Ag ions could directly bind to the active sites of thioredoxin and thioredoxin reductase in *S. aureus*, leading to oligomerization and functional impairment (**Figure** **3**A).^[^ ^70^ ^]^ This disrupted the thiol‐oxidation redox homeostasis used by bacteria to resist oxidative stress. Ag^+^ could also deactivate the respiratory enzymes in the cytoplasm, thereby terminating ATP production and inducing ROS generation to eradicate bacterial cells.^[^ ^71^ ^]^ Cu^2+^ can bind to sulfur groups present in glutathione (GSH), ascorbic acid (AA), and other protein amino acids, leading to protein dysfunction and inhibition of ATP production, impairing DNA replication, and ultimately triggering cell apoptosis (Figure 3B).^[^ ^72^, ^73^, ^74^ ^]^

In addition to affecting proteins, Ag ions can weaken the binding between histone‐like nucleoid structuring (H‐NS) protein and DNA, resulting in the dehybridization of double‐stranded DNA.^[^ ^75^ ^]^ Ag ions can inhibit the synthesis of peptidoglycan, thereby disrupting the normal structure of the cell wall. They can interact with phosphorus in DNA, leading to abnormal DNA replication, thereby modulating cell proliferation. In addition, Ag^+^ can also induce ribosome denaturation to inhibit protein synthesis.^[^ ^71^ ^]^ Cu^2+^ can impede the dNTP biosynthesis pathway leading to bacterial imbalance and hindering bacterial growth.^[^ ^76^ ^]^ The anionic Au NPs constructed by Sun et al. could disrupt the conformation of transmembrane proteins while maintaining the integrity of the membrane, affecting the local properties of lipid bilayer and the function of ion channel (Figure 3C).^[^ ^77^ ^]^ This led to efflux of K^+^ that disturbed K^+^ balance, and depolarization of bacterial cell membranes, ultimately resulting in bacterial death.^[^ ^78^ ^]^

### 2.2 Antibacterial Effect Achieved through Generating ROS

ROS, such as superoxide anion radicals (•O_2_^−^), hydroxyl radicals (•OH), and hydrogen peroxide (H_2_O_2_), are endogenously produced within cells.^[^ ^79^ ^]^ Under normal circumstances, ROS can safeguard cells from oxidative stress through intrinsic regulatory mechanisms.^[^ ^80^ ^]^ However, exposure to elevated levels of ROS can induce oxidative stress, resulting in substantial damage to cell membranes, degradation of crucial proteins and nucleic acids, and initiation of lethal stress response cascades that ultimately lead to cell death.^[^ ^81^ ^]^ Additionally, the oxidative stress induced by ROS can also degrade the extracellular polymeric substance matrix comprising proteins, lipids, and cellular DNA, leading to biofilm eradication.^[^ ^82^, ^83^ ^]^ Consequently, ROS may serve as an effective antibacterial agent.^[^ ^84^ ^]^ Group IB metal‐based NMs can achieve antibacterial effects by generating ROS in several ways: modulating ROS‐related enzymes through metabolic reactions, inducing cells to increase ROS production, or direct participating in ROS production in response to endogenous (enzyme‐like activities) or exogenous (photo/sonodynamic performance) stimulus.

#### 2.2.1 ROS Generation Due to Enzyme‐Like Activities

**Figure 4:**
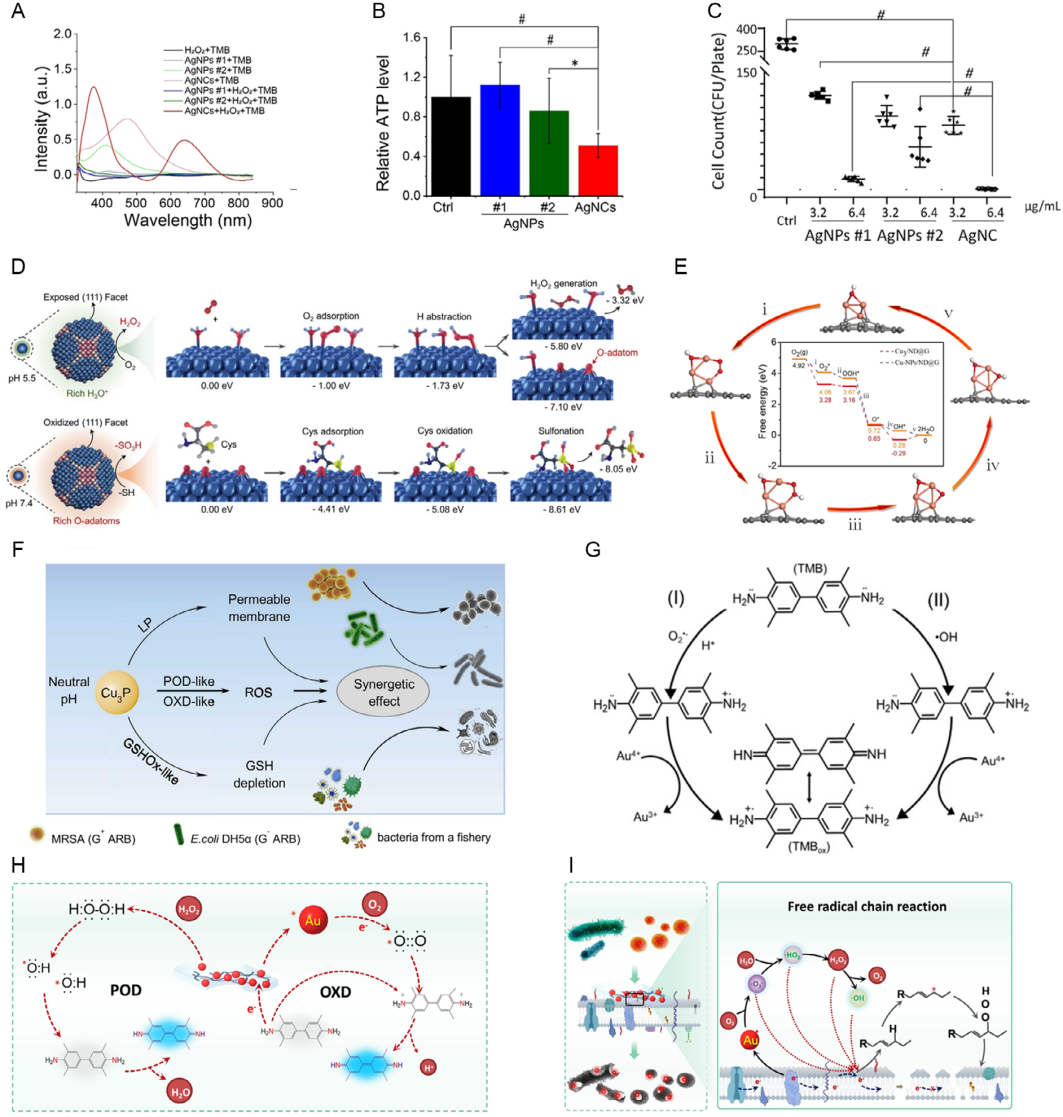
A) Determination of peroxidase‐like properties of Ag NCs and Ag NPs. B) Relative ATP production in *P. aeruginosa* cells upon exposure to Ag NPs and Ag NCs. C) The bactericidal rate of Ag NCs and Ag NPs. Reproduced with permission.^[^ ^86^ ^]^ Copyright 2020, Nature Publishing Group. D) The structural basis and mechanistic studies for the tunable catalytic activities of Ag NCs. Reproduced with permission.^[^ ^87^ ^]^ Copyright 2022, Wiley‐VCH Verlag. E) Theoretical investigation of oxidase‐like activity over Cu‐NPs/ND@G and Cu3/ND@G. Reproduced with permission.^[^ ^90^ ^]^ Copyright 2022, Elsevier. F) Antibacterial mechanism of Cu_3_P NPs. Reproduced with permission.^[^ ^91^ ^]^ Copyright 2022, Elsevier. G) Schematic mechanistic cycles leading to the peroxidase‐ and oxidase‐mimicking oxidation of TMB by the Au^3+^‐NMOFs. Reproduced with permission.^[^ ^97^ ^]^ Copyright 2022, Wiley‐VCH Verlag. H) Mechanism Illustration of the catalytic activities mechanism and I) bactericidal mechanism of Au NPs. Reproduced with permission.^[^ ^98^ ^]^ Copyright 2024, Elsevier.

Some group IB metal‐based NMs exhibit unique enzyme‐like activities, such as peroxidase (POD)‐like activity, which are capable of generating ROS to induce oxidative damage to sulfur‐containing amino acids such as cysteine and methionine, thereby achieving the desired antimicrobial effect.^[^ ^85^ ^]^ Ag nanoclusters (NCs) have been proven to possess POD‐like activity that could reduce the relative ATP production of *P. aeruginosa* by more than half after treatment (**Figure** **4**A,B).^[^ ^86^ ^]^ This was accompanied by outstanding bactericidal effects with a bactericidal rate exceeding 99% at a concentration of 6.40 μg mL^−1^ (Figure 4C). Additionally, Ag NCs demonstrated oxidase (OXD)‐like activity to generate H_2_O_2_ to eliminate bacterial biofilms.^[^ ^87^ ^]^ When Ag NCs enter bacterial cells, they displayed thiol oxidase‐mimetic activity in neutral pH environment of the cytoplasm, preventing the ribosomes from functioning properly and thus inhibiting the development of antibiotic resistance (Figure 4D). Numerous Cu‐based NMs, such as Cu NCs, copper oxide (Cu_*x*_O) NPs, and copper sulfide (CuS) NPs, exhibit enzyme‐like activity and have already been applied in antibacterial research.^[^ ^88^, ^89^ ^]^ Meng et al. developed an atomically dispersed Cu_3_ cluster stabilized on nanodiamond–graphene support and observed that the different oxidation states of Cu (Cu^2+^/Cu^+^/Cu^0^) significantly impact the simulation of enzyme‐like activity (Figure 4E).^[^ ^90^ ^]^ Additionally, Cu_3_P NPs exhibited both OXD‐ and POD‐like activities, capable of producing a large amount of ROS and depleting GSH, showing better antibacterial activity compared to CuO NPs (Figure 4F).^[^ ^91^ ^]^

Au NPs exhibit both OXD‐ and POD‐like activities, but their limited active sites constrain their applications.^[^ ^92^ ^]^ It has been demonstrated that the active sites of Au NPs can be modified and enhanced by adjusting the chemical and geometric environment around the atoms through the use of suitable ligands.^[^ ^93^, ^94^ ^]^ Both Au NPs and Au NCs can attain multiple enzyme‐like activities with different surface modifications.^[^ ^95^, ^96^ ^]^ Willner et al. synthesized Au^3+^‐functionalized UiO‐67 metal–organic frameworks (MOFs) NPs (Au^3+^‐NMOFs) with dual OXD‐ and POD‐like activities, capable of catalyzing the generation of •O_2_^−^ under aerobic conditions or •OH in the presence of H_2_O_2_, thereby exhibiting excellent antibacterial properties (Figure 4G).^[^ ^97^ ^]^ Smaller‐sized materials possess higher specific surface areas, leading to increased atomic utilization and enhanced catalytic performance.^[^ ^98^ ^]^ Zhang et al. restricted the diameter of Au NPs to ≈4.7 nm, resulting in materials with outstanding OXD‐ and POD‐like activities and enhanced antibacterial properties (Figure 4H,I).^[^ ^98^ ^]^

#### 2.2.2 ROS Generation Due to External Stimulation

**Figure 5:**
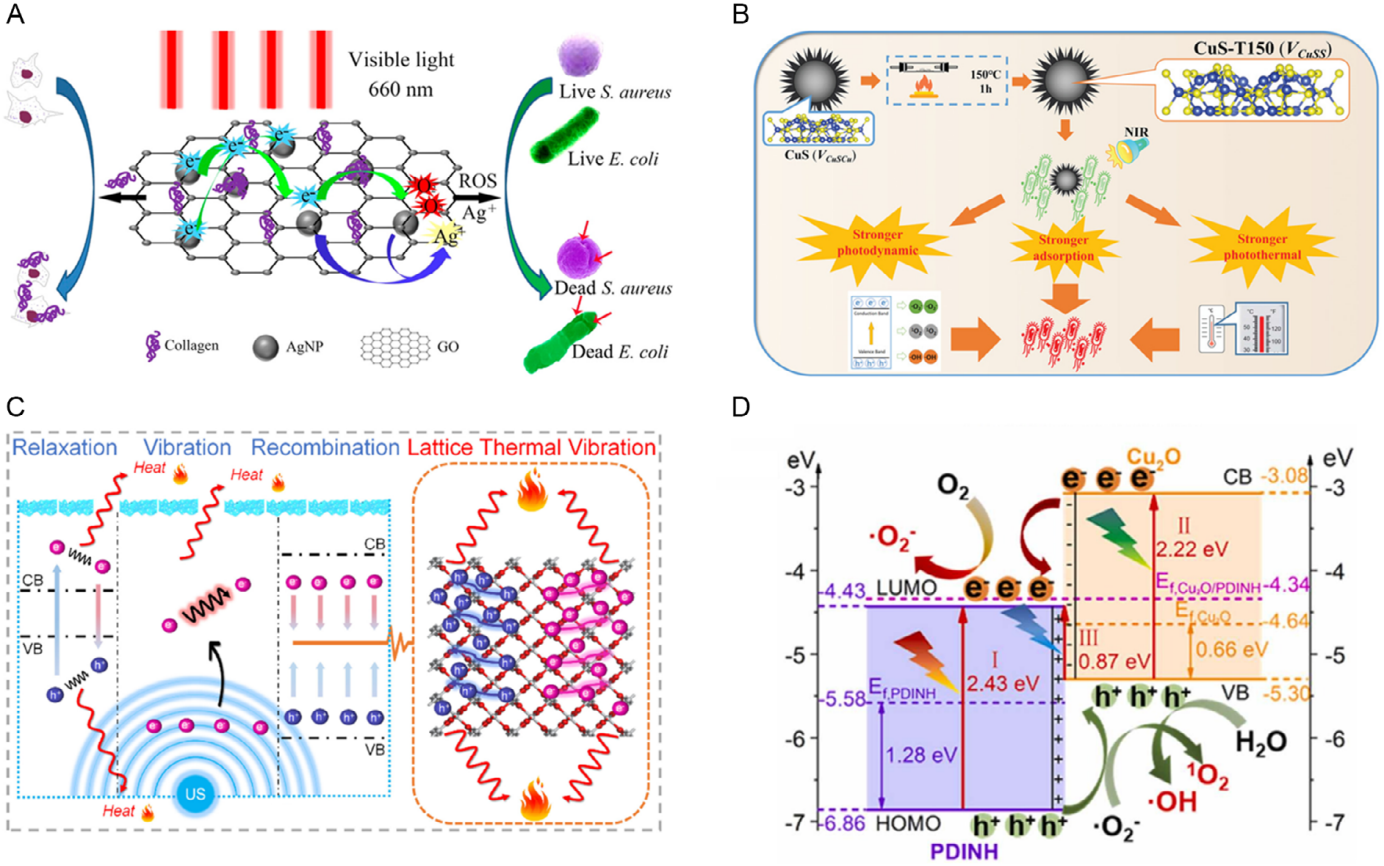
A) Antibacterial action of GO/AgNPs coatings under 660 nm visible light. Reproduced with permission.^[^ ^107^ ^]^ Copyright 2017, American Chemical Society. B) The antibacterial mechanism of CuS against *E. coli* under 808 nm laser irradiation. Reproduced with permission.^[^ ^109^ ^]^ Copyright 2023, John Wiley and Sons Ltd. C) Schematic illustration of the sonothermal mechanism of CuO_2_/TiO_2_ under US. Reproduced with permission.^[^ ^111^ ^]^ Copyright 2023, Elsevier BV. D) Reaction of Cu_2_O/PDINH heterostructure under light. Reproduced with permission.^[^ ^112^ ^]^ Copyright 2023, Elsevier.

In addition to generating ROS through enzyme‐like activity, group IB metal‐based NMs can also produce ROS for antibacterial purposes upon stimulation by external energy fields including optical energy and ultrasound (US). Antibacterial photodynamic therapy (aPDT) utilizes specific wavelengths of light to induce ROS generation, leading to oxidative stress damage to bacteria and subsequent eradication.^[^ ^99^ ^]^ Noble metal NMs, including Ag and Au NPs, exhibit tunable optical properties.^[^ ^100^ ^]^ The localized surface plasmon resonance (LSPR) effect they possess can induce the generation of electron–hole pairs, improve the efficiency of charge carrier separation, thereby enhancing the efficiency of converting light energy into chemical energy.^[^ ^101^, ^102^, ^103^, ^104^ ^]^ For instance, Ag/ZnO and Au/ZnO composites demonstrated enhanced photodynamic performance and antibacterial properties.^[^ ^105^, ^106^ ^]^ Wu et al. obtained Ag NPs‐loaded graphene oxide (GO) nanosheets (NSs) through in situ reduction of Ag with enhanced performance in aPDT.^[^ ^107^ ^]^ Upon light irradiation, the LSPR effect of Ag NPs induced rapid generation and transfer of free electrons to the adjacent GO sheet surface, leading to ROS production and effective bacteria eradication (**Figure** **5**A). The photodynamic action of the GO/AgNPs coatings exhibited rapid and effective bacterial elimination.

CuS NMs have controllable energy band, which can be degraded under near‐infrared (NIR) light along with the production of sulfur vacancies, Cu ions, and carbon residues. During this process, the adsorbed water molecules were converted into hydroxide ions (OH^−^) through the exchange of photoelectrons, and then •OH was produced in the vacancies of the CuS NMs. Additionally, under visible light, O_2_ triggered the charge transfer in CuS NPs in the presence of carbon residues and oxygen residues, promoting the generation of singlet oxygen (^1^O_2_), thereby achieving antibacterial effects.^[^ ^108^ ^]^ Zhang et al. facilitated the separation of electron–hole pairs of CuS nanoballs by modulating vacancies, leading to increased generation of ROS.^[^ ^109^ ^]^ In addition, CuS exhibited enhanced adsorption capacity for O_2_ and elevated photothermal conversion efficiency (PTCE) (Figure 5B). Consequently, the bactericidal rate reached 99.9% under 808 nm laser irradiation. Moreover, Group IB metal‐based NMs can also be integrated with other substances to create Schottky junctions, thereby enhancing catalytic efficacy and ultimately improving their efficacy in aPDT and antibacterial sonodynamic therapy (aSDT).^[^ ^110^ ^]^ aSDT employs low‐intensity US to trigger ROS production for bactericidal effects, exhibiting superior treatment efficacy for deep‐seated bacterial infections due to its enhanced penetration depth in comparison with aPDT. Zhu et al. fabricated a CuO_2_/TiO_2_ heterojunction, in which ultrasonic stimulation induced the release of Cu^2+^ ions from CuO_2_, leading to the generation of oxygen vacancies that reduced the bandgap of TiO_2_ and enhanced carrier migration efficiency, thereby generating ROS.^[^ ^111^ ^]^ Furthermore, US induced extensive cavitation bubble formation through the cavitation effect. Upon bubble collapse, significant energy was released, resulting in the generation of a large number of •OH that synergistically contributed to the bactericidal activity of the material (Figure 5C). Chen et al. constructed a heterojunction comprising Cu_2_O and perylene‐3,4,9,10‐tetracarboximide (PDINH), extending the absorption spectrum from ultraviolet to NIR region, resulting in a 75% increase in light utilization efficiency, enhanced photocatalytic activity, and demonstrated significant bactericidal effects (Figure 5D).^[^ ^112^ ^]^

### 2.3 Antibacterial Mechanism through Photothermal Effect

Au and Ag NPs exhibit a strong LSPR effect, while Cu NPs have relatively longer wavelength LSPR absorption,^[^ ^113^, ^114^, ^115^, ^116^ ^]^ all of which demonstrate excellent PTCE, possessing the potential of PTT. Under the irradiation of a specific wavelength of laser, the LSPR effect induces the conversion of electrons into thermal electrons, facilitating the transfer of light energy to thermal energy, subsequently, achieving bactericidal effects through photothermal mechanisms. Ran et al. synthesized Bi‐Ag NPs, which could generate heat to eradicate bacteria.^[^ ^117^ ^]^ Additionally, the LSPR effect of Bi‐Ag NPs enhanced the electronic transfer between Bi and Ag at the interface, thereby promoting the generation of more photocatalytic ROS and enhancing antibacterial activity through synergistic effects. Hua et al. coated sea urchin‐shaped Bi_2_S_3_ onto Au nanorods (NRs).^[^ ^118^ ^]^ The composite demonstrated outstanding PTCE. Moreover, due to enhanced charge separation efficiency, the core–shell NPs exhibited effective ROS production, collaboratively contributing to its remarkable antibacterial effects. Liu et al. constructed CuS nanoplates (NPLs) with enhanced PTCE, ROS production, and antibacterial performance by adjusting the crystal facets and electronic structures.^[^ ^119^ ^]^

It should be noted that light and heat are involved during the use of PTT; therefore, improper handling can lead to not only bacterial eradication but also unintended damage to normal tissues and potential induction of new inflammation.^[^ ^120^ ^]^ Effective strategies to mitigate this side effect are through targeted delivery and controlled release. For example, the antibacterial NM BCPP composed of protoporphyrin, phenylboric acid (PBA), and BSA@CuS utilizes the bacterial targeting capability of PBA, enabling more precise action at sites of infection.^[^ ^121^ ^]^ This approach reduces the cytotoxicity of CuS to normal cells while enhancing antibacterial efficacy. Moreover, synergistic combinations with other therapies are frequently employed to improve therapeutic effect and minimize adverse effects. Such synergies allow PTT to effectively eliminate bacteria at lower temperatures, reduced dosage of nanoantimicrobial agents, or/and power density of the laser. For example, combining aPDT with PTT can achieve comparable antibacterial efficacy using less ROS and heat compared to PTT or PDT alone.^[^ ^117^, ^118^, ^119^ ^]^ Additionally, integrating aPDT with catalytic material‐mediated therapy can achieve similar results while lowering the therapeutic dose.^[^ ^122^ ^]^

## 3 Antibacterial Properties of Silver‐Based Nanomaterials

Since the emergence of Ag colloids, Ag‐based NMs have been in use for over a century. The high antibacterial activity of Ag‐based NMs has been extensively validated. Furthermore, they have been proven to be nontoxic to humans at trace concentrations, establishing them as an ideal antimicrobial agent.^[^ ^123^ ^]^ Ag‐based NMs including elemental substance, compounds, and composites all exhibit high antimicrobial efficacy, making them one of the most competitive antimicrobial materials.

### 3.1 Nanomaterials of Silver Elemental Substance

**Figure 6:**
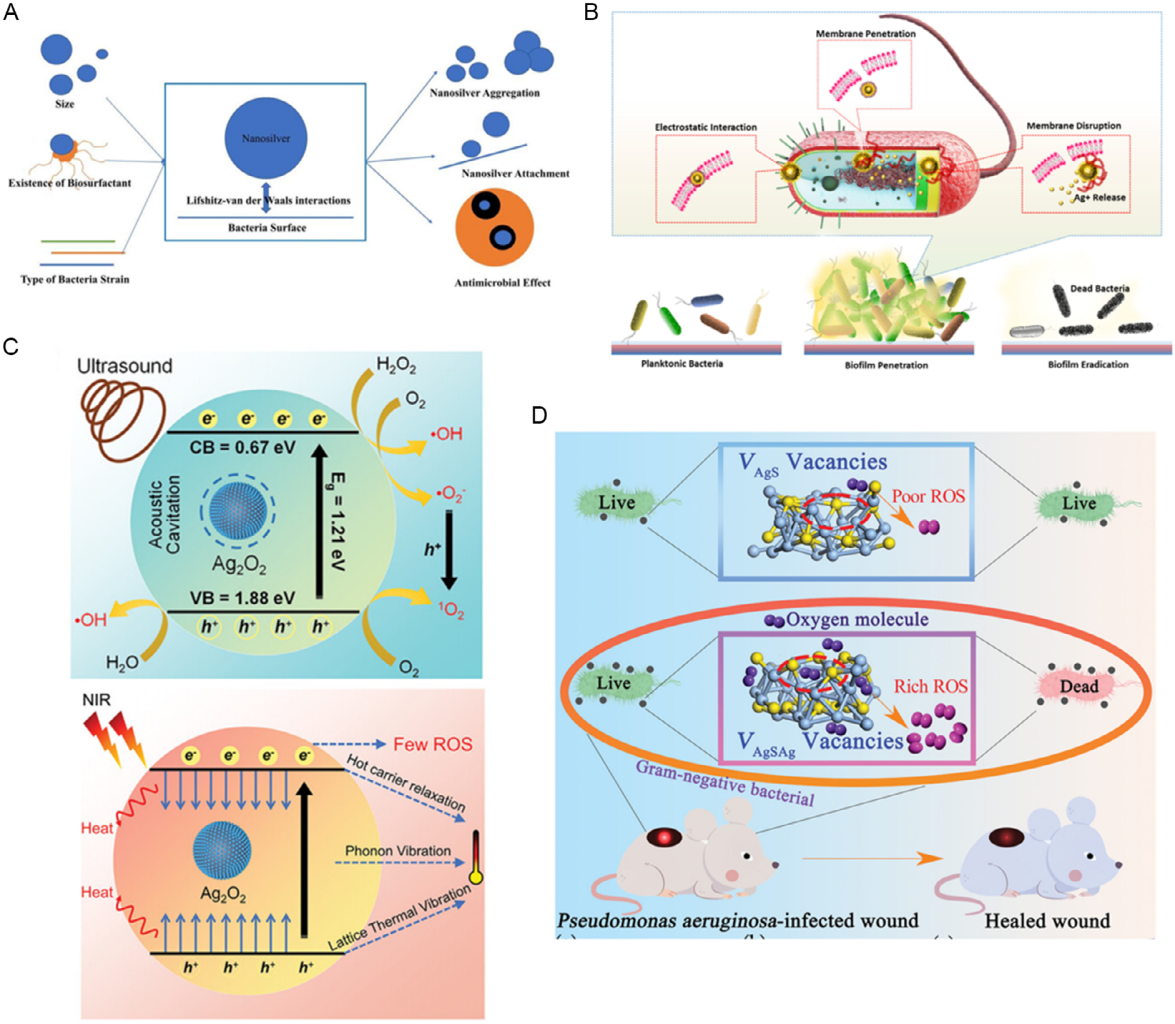
A) Factors influencing the antibacterial effect of nanosilver. Reproduced with permission.^[^ ^124^ ^]^ Copyright 2022, Dove Medical Press Ltd. B) Schematic illustration of the proposed multifaceted bacterial elimination mechanism of pAg NCs. Reproduced with permission.^[^ ^131^ ^]^ Copyright 2022, American Chemical Society. C) The mechanism of ROS production under US irradiation and the photothermal mechanism under NIR irradiation of Ag_2_O_2_ NPs. Reproduced with permission.^[^ ^144^ ^]^ Copyright 2022, Wiley‐VCH Verlag. D) Antibacterial mechanism of Ag_2_S. Reproduced with permission.^[^ ^145^ ^]^ Copyright 2022, Wiley‐VCH Verlag.

The properties of NMs are closely linked to their structures (**Figure** **6**A).^[^ ^124^ ^]^ Consequently, extensive research has been conducted on the impact of morphology and size on the antibacterial performance of Ag‐based NMs to obtain Ag NMs with enhanced antibacterial effects. Compared to other shapes of Ag NPs, the spherical Ag NPs synthesized by Cheon et al. can promote the release of a greater number of silver ions, leading to enhanced antibacterial activity.^[^ ^125^ ^]^ Hu et al. found that triangular NSs with {111} facets exhibited higher antibacterial activity than nanospheres, nanocubes with {100} facets, and nanorods (NRs) featuring both {111} and {100} facets.^[^ ^126^ ^]^ This enhanced activity can be attributed to the greater release of Ag^+^ from the {111} facets and their superior interaction with bacterial surfaces. Furthermore, an increased proportion of high‐atom‐density facets facilitates more effective interactions between Ag NPs and bacteria, thereby enhancing the overall antibacterial efficacy.^[^ ^127^ ^]^ Cubic Ag NPs or plate‐shaped Ag NPs possess better crystal facets compared to quasi‐spherical Ag NPs.^[^ ^128^ ^]^ The presence of angular or edge features in the morphology may lead to physical damage, thereby exhibiting better antibacterial properties. Due to stronger Lifshitz‐van der Waals interactions and larger specific surface areas, smaller Ag NPs exhibit heightened antibacterial properties.^[^ ^124^ ^]^ Furthermore, for Ag NPs with similar size and morphology, a higher density of edges means an increased capacity to induce mechanical damage to bacteria, accordingly increasing their antibacterial efficacy.^[^ ^129^ ^]^

Ultrasmall Ag NCs not only possess a higher surface‐volume ratio and an increased rate of Ag ion release, but also exhibit enhanced capability to penetrate the interior of bacteria for eradication.^[^ ^130^ ^]^ Moreover, the modification of Ag NCs can further enhance their antibacterial performance. For instance, Haidari et al. designed ultrasmall (<3 nm) and highly dispersed polycationic Ag (pAg) NCs, with MIC ranging from 3.375 to 6.75 μg mL^−1^, significantly lower than previously reported Ag NPs and Ag NCs with similar sizes (Figure 6B).^[^ ^131^ ^]^ To address the challenge of poor stability and hindered reactivity of Ag NCs under physiological conditions caused by spontaneous aggregation and rapid oxidation, Lin et al. precisely controlled the overall and grain size of Ag NCs using a single‐molecule micelle template and synthesized Ag@Template NCs.^[^ ^132^ ^]^ It exhibited remarkable antibacterial activity with almost no cytotoxicity, thus achieving long‐term antibacterial performance.

In addition to 0D NPs, extensive research has also been conducted on other shapes of Ag‐based NMs including 1D Ag nanowires (NWs) and 2D Ag NPLs or NSs. Compared to Ag NPs, the intrinsic antibacterial activity of Ag NWs exhibits no statistically significant difference, suggesting that the morphology of Ag NWs has minimal impact on their antibacterial performance. However, their antibacterial activity can be enhanced by modulation of the release rate of Ag ions through strategies such as thermal treatment or electron beam irradiation.^[^ ^133^, ^134^, ^135^ ^]^ Furthermore, Ag NWs are commonly used to form membranes or network structures to capture and kill bacteria.^[^ ^136^ ^]^ Due to their high aspect ratio, 2D Ag‐based NMs possess a larger specific surface area compared to Ag NPs of the same volume, demonstrating superior bactericidal activity.^[^ ^137^, ^138^ ^]^

### 3.2 Nanomaterials of Silver Compound

Silver compounds can release Ag ions and act as semiconductor catalysts to generate ROS in response to external stimuli, thereby exhibiting excellent antibacterial performance. Silver oxides, including Ag_2_O and AgO, exhibit significant intrinsic bactericidal effects, among which, Ag_2_O possesses strong broad‐spectral antibacterial properties as a p‐type semiconductor material.^[^ ^139^, ^140^, ^141^, ^142^, ^143^ ^]^ Additionally, as a metal peroxide, Ag_2_O_2_ possess inherent physical and chemical properties that enable the generation of H_2_O_2_, making them suitable for various biomedical applications.^[^ ^144^ ^]^ Furthermore, Ag_2_O_2_ can release Ag^+^ and ROS upon external stimuli such as US and NIR light. It demonstrates exceptional PTCE under NIR light irradiation, thereby exhibiting strong antibacterial and antibiofilm capabilities (Figure 6C).

Ag_2_S is a semiconductor with a bandgap of ≈0.9–1.05 eV. Due to its photothermal and photocatalytic properties, Ag_2_S is often used as a photosensitizer for aPDT. To address the issue of low PTCE that leads to diminished carrier concentration and ROS production, Kim et al. synthesized two types of Ag_2_S NCs with different vacancies by adjusting the surface electronic structure through vacancy engineering (Figure 6D).^[^ ^145^ ^]^ This modification altered physicochemical properties of Ag_2_S NCs, enabling them to exhibit excellent antibacterial activity even in the absence of light. Silver halides (AgX, X = Cl, Br, I) can also release Ag ions and generate electron–hole pairs under light exposure, making them effective photocatalysts for antibacterial treatment.^[^ ^146^, ^147^, ^148^, ^149^ ^]^

## 4 Antibacterial Properties of Copper‐Based Nanomaterials

Similar to Ag, Cu‐based NMs have a long history in antibacterial applications.^[^ ^150^ ^]^ As an essential element for the human body, Cu is involved in various biological processes, including respiration, metabolism, and cell signaling, and has better biocompatibility compared to Ag.^[^ ^151^ ^]^ In 2008, Cu was recognized by the United States EPA as the first metal antibacterial agent.^[^ ^50^, ^51^ ^]^ Cu‐based NMs, including elemental substance, oxides, and sulfides, have been widely studied for their excellent antibacterial activity and good biocompatibility.

### 4.1 Nanomaterials of Copper Elemental Substance

The antibacterial efficacy of Cu‐based NMs is typically influenced by the quantity of released Cu ions, with a higher uptake of Cu ions by bacteria correlating to an enhanced antibacterial effect.^[^ ^152^ ^]^ Lan et al. prepared Cu NMs with different morphologies including NWs, NSs, and nanoflowers (NFs) and studied their antibacterial properties.^[^ ^153^ ^]^ The results substantiated the excellent antibacterial properties of hydrophobic surfaces attributed to the release of Cu ions. Among the three morphologies, Cu NWs exhibited the highest capacity in penetrating bacterial cells, directly causing bacterial lysis, and thus demonstrated the best antibacterial performance.

**Figure 7:**
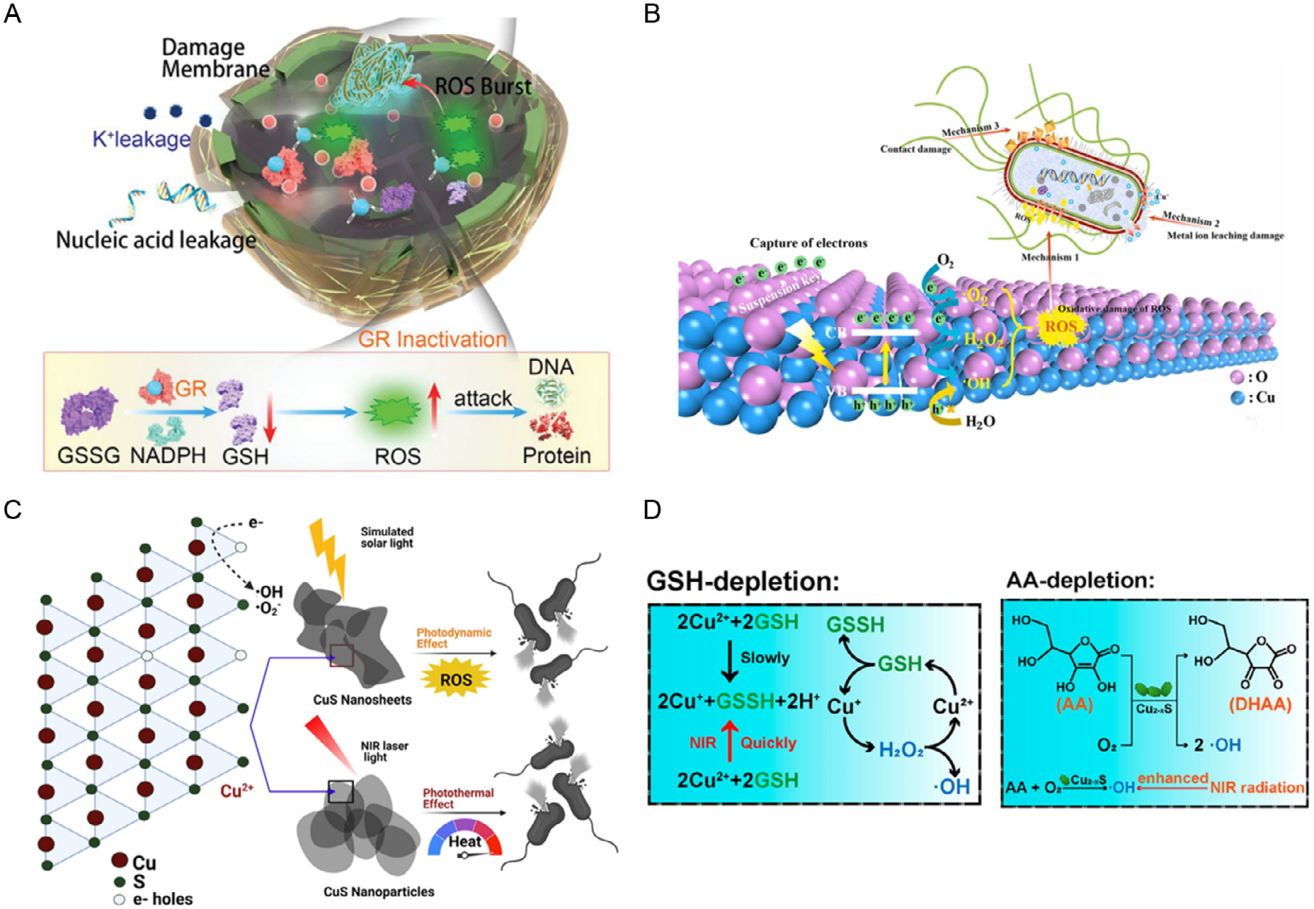
A) Schematic illustration of the action mechanisms of CuCs on methicillin‐resistant *S. aureus* (MRSA). Reproduced with permission.^[^ ^155^ ^]^ Copyright 2021, Wiley‐VCH Verlag. B) Antibacterial mechanism of Vo–(111) Cu_2_O photocatalyst. Reproduced with permission.^[^ ^161^ ^]^ Copyright 2023, Elsevier. C) Schematic illustration of photodynamic and photothermal antibacterial pathways mediated by CuS NSs and NPs. Reproduced with permission.^[^ ^108^ ^]^ Copyright 2022, Academic Press Inc. D) Illustration of the POD‐mimic catalytic process and the GSH depletion process. Reproduced with permission.^[^ ^166^ ^]^ Copyright 2021, American Chemical Society.

Cu NCs in antibacterial applications have the advantages of being cost‐effective and have shown excellent broad‐spectrum antibacterial activity.^[^ ^154^ ^]^ Lu et al. constructed Cu NCs using theanine peptides, which exhibited wide‐spectrum antibacterial capabilities at extremely low doses in vitro.^[^ ^155^ ^]^ Cu NCs could disrupt the bacterial cell wall structure and inhibit GSH reductase activity, leading to an imbalance in the GSH/oxidized GSH (GSSG) ratio, which generated excess ROS to attack DNA and accelerate bacterial cell wall destruction, ultimately resulting in bacterial death (**Figure** **7**A). The theanine peptide‐based Cu NCs exhibited enhanced stability, with no release of Cu ions observed within 30 days, thereby demonstrating significantly reduced cytotoxicity to normal mammalian cells compared to Ag NCs.

### 4.2 Nanomaterials of Copper Compound

Cu compound NPs possess photothermal, photodynamic, catalytic activities, and intrinsic ion toxicity, making them suitable for various biomedical applications, particularly showing immense potential in the antibacterial field.^[^ ^156^ ^]^ Among the Cu_*x*_O, CuO, and Cu_2_O are semiconductors with superior catalytic properties. CuO NPs can generate substantial amounts of ROS, inducing oxidative stress responses that interfere with the phosphates‐containing functional groups in proteins and sulfur‐containing functional groups in DNA molecules, ultimately leading to the disruption of DNA, proteins, and the electron transport chain.^[^ ^157^, ^158^, ^159^ ^]^ In addition, the electrostatic force and nanoporous structure of CuO NPs also enable effective interaction with bacterial cellulose for antibacterial effects.^[^ ^158^ ^]^ In contrast to CuO NPs, Cu_2_O NPs can absorb visible light to produce ROS, but due to its propensity of electron–hole pair recombination, the ROS produced by Cu_2_O alone are insufficient for bactericidal activity.^[^ ^160^ ^]^ Wang et al. found that the antibacterial performance of Cu_2_O NPs varied with the exposed crystal planes. Cu_2_O NPs with exposed (111) planes exhibited the best antibacterial performance compared to those with exposed (100) and (110) planes.^[^ ^161^ ^]^ This is resulted from the combination of oxidative damage induced by ROS under light irradiation, ion dissolution, and contact damage (Figure 7B). As a Fenton‐type metal peroxide, CuO_2_ can generate ROS to induce oxidative stress responses within cells, thereby exerting bactericidal effects. Additionally, it exhibits promising therapeutic potential in promoting wound healing.^[^ ^162^, ^163^ ^]^

CuS NPs exhibit excellent photocatalytic and photothermal properties with controllable energy band structure, making them highly promising for antibacterial applications.^[^ ^164^, ^165^ ^]^ Kuo et al. synthesized CuS microspheres (MSs), NSs, and NPs and compared the effects of morphology on their antibacterial performance.^[^ ^108^ ^]^ Under simulated sunlight, all three forms of CuS could produce photoelectrons and release Cu ions, which reacted with atmospheric moisture to generate •OH and •O_2_^−^, leading to bacterial death (Figure 7C). Besides, CuS NPs were more effective than MSs and NSs in generating heat and antibacterial activity under NIR laser irradiation due to their smaller size. Zhou et al. employed quantum mechanics to theoretically demonstrate that sulfur vacancies could alter the electronic structure of Cu active sites, lowering the reaction energy barrier from H_2_O_2_ to •OH, thereby enhancing POD‐like activity.^[^ ^166^ ^]^ The optimized CuS NPs effectively consumed GSH and AA, improving antibacterial performance and achieving synergistic effects with PTT under NIR light irradiation (Figure 7D), resulting in a bactericidal rate of over 99% against both *E. coli* and *S. aureus*. Additionally, Chen et al. synthesized ≈5 nm‐sized CuS NCs, which exhibited excellent antibacterial performance, completely killing bacteria at a dose of 76.8 μg mL^−1^.^[^ ^167^ ^]^

## 5 Antibacterial Properties of Gold‐Based Nanomaterials

Au NMs possess excellent stability, facile modification, and a high surface‐area‐to‐volume ratio, making them highly desirable for use in the biomedical field in recent years.^[^ ^168^, ^169^ ^]^ Au NMs without modification mainly exist as elemental substance, and their performance is less dependent on Au ions, exhibiting relatively weaker antibacterial performance compared to Ag and Cu. Au NPs can attach to the microbial cell wall via surface charges to disrupt the bacterial cell membrane to exert antibacterial effects. As one of the noble metals, Au NPs also exhibit significant LSPR properties, enabling the conversion of light energy into thermal energy.^[^ ^113^, ^114^ ^]^ Additionally, Au NPs possess tunable surface characteristics and unique optical properties. Therefore, Au NMs have been applied in aPDT and PTT in addition to causing bacterial death through ROS‐induced oxidative stress.^[^ ^170^, ^171^ ^]^

**Figure 8:**
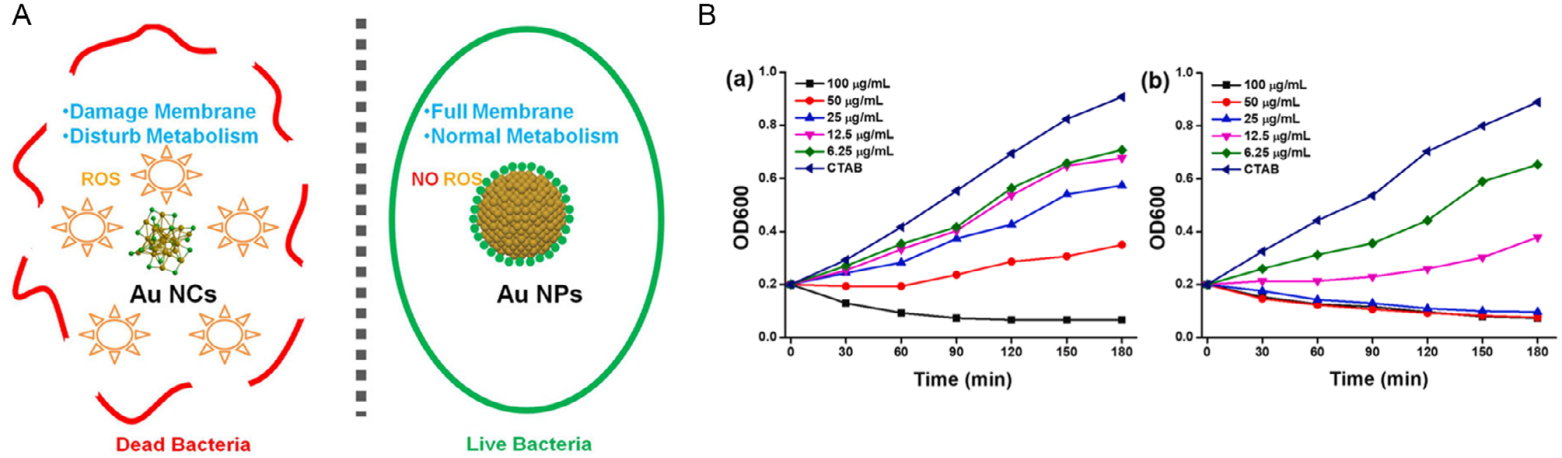
A) The difference in antibacterial activity between Au NPs and Au NCs. Reproduced with permission.^[^ ^172^ ^]^ Copyright 2017, American Chemical Society. B) The PTT property of Au NRs (left) and Au NBPs (right). Reproduced with permission.^[^ ^174^ ^]^ Copyright 2021, Elsevier.

Xie et al. discovered that reducing the size of Au NMs (typically <2 nm) resulted in broad‐spectrum antibacterial activity.^[^ ^172^ ^]^ Due to their ultrasmall size, Au NCs could be easily internalized, subsequently inducing metabolic imbalances in bacteria and increasing intracellular ROS to kill bacteria (**Figure** **8**A). Au NCs could also damage the bacterial cell membrane, impacting genes involved in transcription and translation. Additionally, the LSPR and catalytic properties can be further optimized through manipulating morphology of Au NMs.^[^ ^173^ ^]^ Sibidou et al. synthesized Au NRs with a (200) plane and Au nanobipyramids (NBPs) with a (111) plane as photothermal agents for noninvasive PTT.^[^ ^174^ ^]^ Density functional theory simulations revealed that the surface adsorption energy of Au (111) was lower than that of Au (100), making it easier to desorb from the Au (111) surface for photothermal effects. Therefore, Au NBPs demonstrated better heat dissipation. Experiments validated their superior photothermal performance and antibacterial efficacy in comparison to Au NRs (Figure 8B).

## 6 Antibacterial Properties of Group IB Metal‐Based Nanocomposites

Although group IB metal‐based NMs exhibit excellent antibacterial performance through various mechanisms, there remains a need to enhance their antibacterial activity to meet the challenges posed by complex bacterial infections and application requirements. The high surface energy of group IB metal‐based NMs often leads to aggregation and deposition, which diminish their antibacterial efficacy. To address this, carrier materials with good biocompatibility can be employed to stabilize the group IB metal‐based NMs and achieve controlled ion release while reducing their toxicity. Additionally, rational design can be adopted to enhance the photothermal and photodynamic properties of group IB metal‐based NMs, thereby improving their antibacterial performance. Moreover, combining group IB metal‐based NMs with other components possessing antibacterial activity can also lead to the development of nanocomposites with superior antibacterial effects or novel antibacterial mechanisms.

### 6.1 Metal‐Based Nanocomposites

When group IB metal‐based NMs are combined with other metals to form composite materials, the band structure of the material can be optimized to enhance electron transfer efficiency, leading to enhanced photocatalytic or photothermal performance and thereby achieving increased antibacterial effects. One of the most prevalent applications involves creating heterostructures through the combination of group IB metal‐based NMs with other metals. In this context, the LSPR effect can significantly improve electron transfer efficiency, ultimately enhancing antibacterial effects through photocatalytic reactions under light.^[^ ^175^ ^]^

**Figure 9:**
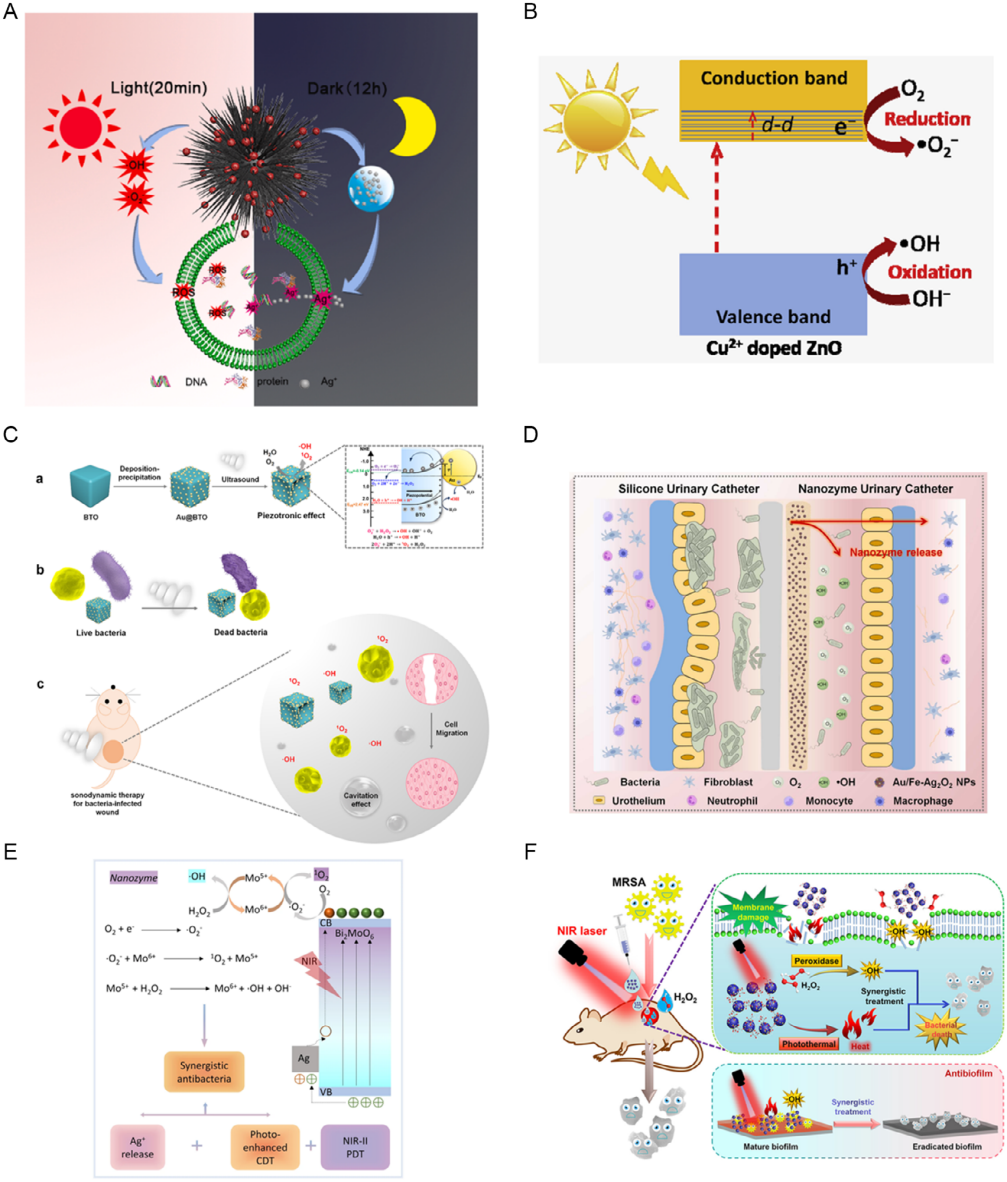
A) Schematic illustration of the possible mechanisms of the photocatalysis, physical puncture and Ag release of BU‐TiO_2−*X*_/Ag_3_PO_4_ heterostructure. Reproduced with permission.^[^ ^176^ ^]^ Copyright 2021, KeAi Communications Co. B) A proposed photocatalytic mechanism of Cu^2+^‐doped ZnO under simulated solar light irradiation. Reproduced with permission.^[^ ^177^ ^]^ Copyright 2020, Elsevier Ltd. C) Schematic illustration of sonodynamic bacterial elimination of piezoelectric nanocomposites Au@BTO. Reproduced with permission.^[^ ^181^ ^]^ Copyright 2021, Elsevier. D) Action mechanism of the nanozyme(Au/Fe–Ag_2_O_2_ NPs)‐reinforced urinary catheter. Reproduced with permission.^[^ ^182^ ^]^ Copyright 2024, Elsevier. E) Schematic illustration of the NIR‐enhanced catalytic activity of Ag/Bi_2_MoO_6_ nanozyme for synergistic bacterial therapy. Reproduced with permission.^[^ ^186^ ^]^ Copyright 2023, Springer Nature. F) Schematic illustration of the eradication of MRSA infection through treatment with a gold‐ Au‐doped MoO_3–*x*_ hybrid. Reproduced with permission.^[^ ^191^ ^]^ Copyright 2022, American Chemical Society.

Xu et al. fabricated a BU‐TiO_2−*X*_/Ag_3_PO_4_ (3:1) heterojunction with a narrow bandgap and high efficiency in separating photogenerated electron–hole pairs, thereby enhancing the photocatalytic performance of Ag_3_PO_4_.^[^ ^176^ ^]^ Additionally, the nanospikes on the surface of BU‐TiO_2−*X*_ could physically puncture bacterial cells, causing mechanical damage to the bacteria (**Figure** **9**A). Moreover, oxygen vacancies on BU‐TiO_2−*X*_ inhibited the reduction of Ag^+^ to Ag^0^, thus reducing its toxicity and improving its photostability. This heterojunction exhibited significant antibacterial effects of 99.76 ± 0.15% against *E. coli* and 99.85 ± 0.09% against *S. aureus* after 20 min of light exposure followed by 12 h of dark incubation. Qi et al. developed Cu‐doped ZnO NPs for antibacterial treatment.^[^ ^177^ ^]^ The Cu doping altered the bandgap structure of ZnO and provided more dopant states, allowing photogenerated electrons to easily transfer from the valence band of ZnO to the localized Cu energy levels and between the energy levels of Cu dopant via *d–d* transitions (Figure 9B). This increased the separation efficiency of photogenerated electrons and holes and significantly inhibited their recombination and enhanced photocatalytic activity, thereby exhibiting a pronounced antibacterial effect under simulated sunlight. Chen et al. coupled Ag NPs with Cu_*x*_O and SrTiO_3_ to construct a Z‐scheme heterojunction.^[^ ^178^ ^]^ The synergistic effect of the SPR effect from Ag NPs and the Z‐scheme heterojunction resulted in excellent antibacterial properties. Francis et al. combined p‐type semiconductor CuO with n‐type semiconductor AgX, constructing CuO/AgX (X = Cl, Br, I) nanocomposites that exhibited excellent antibacterial performance under both light and dark conditions.^[^ ^179^ ^]^ Feng et al. modified Cu_2_O with Ag NPs. The SPR effect of Ag NPs and the Schottky barrier between the two materials facilitated the separation of electron–hole pairs, improving photocatalytic efficiency.^[^ ^180^ ^]^ The antibacterial efficacies against *S. aureus* and *P. aeruginosa* was 99% and 98%, respectively, demonstrating superior long‐term antibacterial activity over a period of 60 days.

In addition to the improvement of their photocatalytic and photothermal characteristics, the constructed heterojunctions can also facilitate ROS generation under US excitation or optimize their own catalytic activity for antibacterial applications. Wu et al. chemically reduced Au NPs onto the surface of BaTiO_3_ (BTO) to form Au@BTO Schottky junctions for antibacterial applications.^[^ ^181^ ^]^ The Au NPs induced band bending in BTO, promoting the separation and migration of electron–hole pairs. Under US induction, the surface piezoelectric potential facilitated interactions between polarized charges/charge carriers and surrounding molecules (H_2_O and O_2_) to produce ROS through redox reactions, ultimately achieving antibacterial effects (Figure 9C). With 4 min of US irradiation, the antibacterial rate can reach 99.94%. Zhu et al. constructed an Au/Ag_2_O_2_ Schottky junction, where Ag_2_O_2_ was employed for autonomous O_2_ supply and Ag ion release, iron was incorporated and used as an active site, and the dopant of Au NPs formed the Schottky heterojunction, thereby achieving POD‐ and catalase‐like activities with robust antibacterial performance, resulting in a 99.999% bactericidal rate (Figure 9D).^[^ ^182^ ^]^ Li et al. employed Ag to facilitate the transfer of electrons and holes in Cu_2_O, thereby enhancing the stability and performance of Cu_2_O and establishing a semiconductor heterojunction of Cu_2_O/Ag.^[^ ^183^ ^]^ In comparison with pure Cu_2_O, Cu_2_O/Ag demonstrated increased generation of ROS and superior antibacterial efficacy.

Except for forming semiconductor heterojunctions, group IB metal‐based NMs can act as cocatalysts to prevent the rapid recombination of electron–hole pairs.^[^ ^184^ ^]^ Moreover, doping low concentrations of group IB metal‐based NMs into other photocatalytic materials can significantly improve their performance.^[^ ^185^ ^]^ Cao et al. optimized the charge separation performance of Bi_2_MoO_6_ by leveraging Ag as a plasmonic metal with a complex permittivity, which increased the absorption in stronger absorption in the NIR‐II region and enhanced POD‐like activity for ^1^O_2_ generation, resulting in a bactericidal rate that exceeded 99% (Figure 9E).^[^ ^186^ ^]^ Zhu et al. enhanced the photocatalytic activity of MoS_2_ using the conductivity and SPR effect of Ag NPs, thereby bolstering its antibacterial performance under visible light.^[^ ^187^ ^]^ Luque et al. decorated CeO_2_ with Ag NPs to narrow down its bandgap, leading to the production of more free radicals and stronger antibacterial activity.^[^ ^188^ ^]^ Wang et al. combined the self‐supplied H_2_O_2_ activation of the Fenton‐like reaction by CuO_2_ with the photothermal effects of WS_2_ NFs for synergistic antibacterial effects, enhancing both antibacterial performance and biocompatibility to promote wound healing.^[^ ^189^ ^]^ Yi et al. utilized the electron capture capability of Au NRs to enhance electron transfer in zirconium‐based porphyrin MOFs (HNTMs), improving the separation efficiency of electron–hole pairs and thus boosting the sonocatalytic activity of red cell membrane (RBC)‐HNTM‐Pt@Au.^[^ ^190^ ^]^ In addition to their electron capture capability, Au NRs also enhanced ultrasonic cavitation and improved the absorption of US energy in the system, increasing ROS production. The RBC‐HNTM‐Pt@Au exhibited an antibacterial rate of 99.9% after 15 min of US stimulation and successfully cured tibial osteomyelitis in rats with negligible bone loss. Cao et al. integrated Au into MoO that enhanced the PTCE of the material by ≈12.3% and augmented its POD‐like activity due to the LSPR effect of Au (Figure 9F).^[^ ^191^ ^]^ The bactericidal rate reached 99.76% due to synergistic effects of increased •OH and thermal energy. Wu et al. prepared enzyme‐like bimetallic oxides of Cu and Mn, forming Cu_1.5_Mn_1.5_O_4_ cage‐like framework nanospheres.^[^ ^192^ ^]^ The spherical structure increased the exposure of active edge sites and exhibited enhanced OXD‐, POD‐, and GSH peroxidase (GPx)‐like activities. These enzyme‐like activities led to the generation of substantial amounts of •OH while depleting GSH at the infected site and reducing the consumption of ROS, thereby conferring significant antibacterial properties.

### 6.2 Inorganic Nonmetal‐Based Nanocomposites

**Figure 10:**
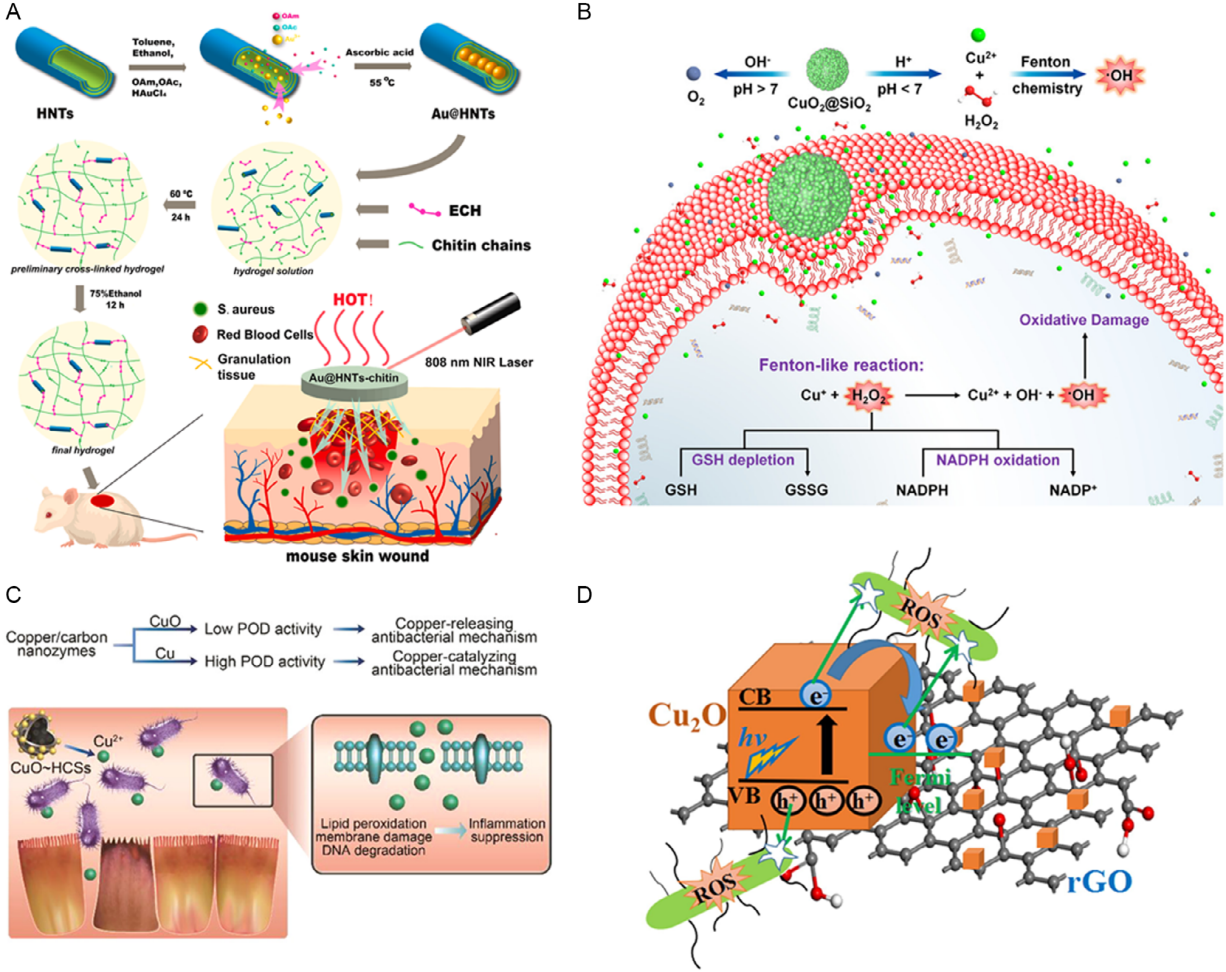
A) The preparation process and application of Au@HNTs‐chitin hydrogel. Reproduced with permission.^[^ ^193^ ^]^ Copyright 2023, KeAi Communications Co. B) pH‐responsive antimicrobial Fenton nanosystem of CuO_2_@SiO_2_ for ROS Production. Reproduced with permission.^[^ ^197^ ^]^ Copyright 2021, American Chemical Society. C) Antibacterial mechanism of Cu/C nanomases. Reproduced with permission.^[^ ^201^ ^]^ Copyright 2019, American Chemical Society. D) Mechanism of ROS production of rGO–Cu_2_O nanocomposites. Reproduced with permission.^[^ ^204^ ^]^ Copyright 2019, Academic Press Inc.

Inorganic nonmetallic NMs with large specific surface areas or large pore sizes can serve as ideal carriers to address the challenges of rapid release, facile aggregation, and short‐term degradation of IB metal‐based NMs. For example, Zhao et al. controlled the particle size of Au NPs to 5–10 nm and prevented its aggregation by filling Au NPs into the cavities of halloysite nanotubes (HNTs), resulting in enhanced catalytic efficiency of Au@HNTs compared to Au NPs (**Figure** **10**A).^[^ ^193^ ^]^ The photothermal properties of small‐sized Au NPs further facilitated Au@HNTs in exhibiting excellent antibacterial effects. Liu et al. used phosphate‐based glass (PBG) to synthesize a biodegradable Cu‐doped nanocatalyst (Cu‐PBG), which exhibited POD‐like activity in acidic wounds.^[^ ^194^ ^]^ Cu‐PBG could gradually degrade in physiological environments, and release Cu ions, thus delivering satisfactory antibacterial effects while remaining biodegradable. NMs with large specific surface areas and porous structures, such as nanoporous carbon nitride and carbon nanotubes, provide excellent carriers for Ag NPs.^[^ ^195^, ^196^ ^]^ Their porous morphology could enhance bacterial adsorption efficiency and facilitate the penetration of the composites into bacterial membranes, thereby improving bactericidal activity. Li et al. encapsulated CuO_2_ with silica nanospheres to form mesoporous CuO_2_@SiO_2_, which efficiently and sustainably generated ROS in acidic environments, achieving a 99.99% bactericidal rate with good cell compatibility (Figure 10B).^[^ ^197^ ^]^ Similarly, metal graphitic nanocapsules were synthesized by encapsulating group IB metal‐based NMs within graphite shells.^[^ ^198^ ^]^ The metal graphitic nanocapsules demonstrated exceptional stability, and the NIR light absorption capability of the graphite shell can synergistically enhance their antibacterial performance.

Inorganic nonmetallic NMs, in addition to serving as carriers, can enhance the antibacterial performance of group IB metal‐based NMs through their own photothermal/photocatalytic properties or enzyme‐like activities. Vidyasagar et al. utilized graphitic carbon nitride (g‐C_3_N_4_) to construct Ag/AgO/g‐C_3_N_4_ heterojunctions as visible light photocatalysts for antibacteria.^[^ ^199^ ^]^ By leveraging the SPR effect of Ag and the heterojunction structure, Ag/AgO/g‐C_3_N_4_ exhibited enhanced light absorption and improved charge carrier separation, resulting in excellent antibacterial performance. Bai et al. synthesized plasmon‐enhanced nanozymes (CuS@GDY) consisting of graphdiyne (GDY) nanowalls encapsulating hollow CuS nanocubes.^[^ ^200^ ^]^ The narrow bandgap of GDY formed a p–p heterojunction that improved light absorption and the LSPR effect of CuS promoted plasmon–exciton coupling under NIR light irradiation. This synergistic effect resulted in photothermal performance and enhanced POD‐like activity, achieving an antibacterial efficacy of over 99.999%. Xi et al. designed two Cu/C nanozymes by anchoring a carbon framework to physically prevent the aggregation of Cu NPs and improve their catalytic activity.^[^ ^201^ ^]^ The enzyme‐like activity and antibacterial ability of Cu/C nanozymes could be modulated by adjusting the oxidation state of Cu. The results showed that nanozyme with Cu^0^ exhibited higher enzyme‐like activity compared to the nanozyme with Cu^2+^ and thus generated ROS for antibacterial purposes, while nanocomposite with low POD‐like activity could release Cu^2+^ to exert significant antibacterial effects (Figure 10C). Wang et al. employed 2D MXene, known for its exceptional solar energy conversion efficiency, in conjunction with CuS to create Ti_3_C_2_@CuS.^[^ ^202^ ^]^ It showed enhanced thermal stability and improved separation efficiency of electron–hole pairs in CuS, resulting in increased production of ROS for antibacterial purposes. Ti_3_C_2_@CuS showed excellent long‐term antibacterial performance, maintaining a bactericidal efficacy of over 90% for more than 30 days.

2D NMs graphene and its derivatives possess large surface areas and excellent electrical conductivity, making them ideal drug carriers for IB metal‐based NMs with outstanding antibacterial properties.^[^ ^203^ ^]^ Yang et al. prepared stable rGO–Cu_2_O nanocomposites utilizing the interaction of Cu_2_O with functional groups on the rGO basal plane.^[^ ^204^ ^]^ The rGO could create a protective barrier for Cu_2_O dispersing, preventing the rapid leaching of Cu ions. Additionally, rGO promoted the separation of photoexcited carriers in Cu_2_O NPs, promoting ROS generation and prolonging their lifespan. The edges of rGO NSs could cause physical damage to bacterial cell membranes, facilitating the interaction between Cu ions and bacteria (Figure 10D). The synergistic antibacterial mechanisms of rGO–Cu_2_O resulted in sustained antibacterial effects, with antibacterial activity 35% higher than that of pure Cu_2_O after 30 days. Meng et al. anchored Cu atoms by constructing Cu—C bonds between defect‐rich GO surfaces and Cu atoms, resulting in atomic‐level dispersed and fully exposed Cu_3_ clusters as active centers stabilized on defect‐rich nanodiamond–graphene hybrid carriers.^[^ ^90^ ^]^ The synergistic interaction between adjacent Cu atoms in each cluster significantly enhanced OXD‐like activity, achieving a 100% antibacterial rate. Tu et al. utilized cation–π interactions that attract a substantial quantity of Cu^2+^ onto rGO to produce rGO–Cu nanocomposites.^[^ ^64^ ^]^ The charge interactions drove the rapid transfer of rGO–Cu and assembly onto bacterial cells, demonstrating significant selective antibacterial activity. During this process, Cu^2+^ was reduced to Cu^0^, enhancing antibacterial activity while maintaining very low surrounding Cu‐ion concentrations (less than 0.5 μM), posing no toxicity to mammalian cells. Fathalipour et al. used silane ligands as modifiers to prevent the aggregation of GO NSs and then used it as a substrate and stabilizer to produce a high‐density Ag NPs layer of ≈8 nm, thereby efficiently enhancing antibacterial activity and resulting in significantly enhanced antibacterial activity.^[^ ^205^ ^]^

### 6.3 Organic‐Based Nanocomposites

**Figure 11:**
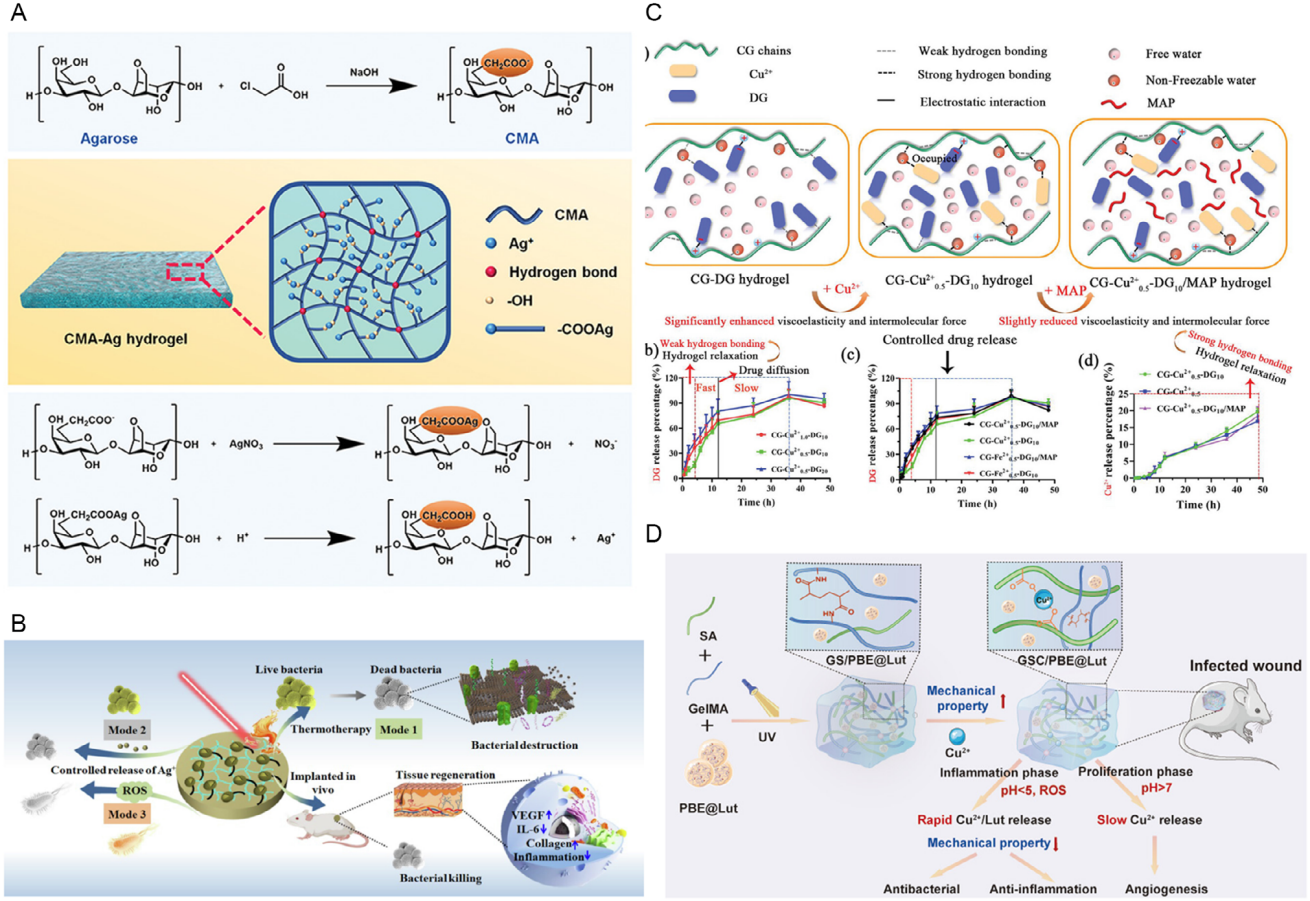
A) The pH‐responsive release and architecture of Ag^+^ from the hydrogels. Reproduced with permission.^[^ ^216^ ^]^ Copyright 2020, Wiley‐VCH Verlag. B) Schematic diagram of trimodal bacterial killing strategy and in vivo tissue regeneration characteristics of the composite Ag_2_S quantum dot/mSiO_2_ NPs hydrogel. Reproduced with permission.^[^ ^218^ ^]^ Copyright 2022, Elsevier BV. C) The mode and strength of intermolecular interactions in different hydrogels. Reproduced with permission.^[^ ^204^ ^]^ Copyright 2024, Wiley‐VCH Verlag. D) The self‐regulation mechanism of hydrogels under different pH conditions. Reproduced with permission.^[^ ^220^ ^]^ Copyright 2024, American Chemical Society.

Polymeric NMs, serving as versatile nanocarriers, can provide sustained release and enable externally triggered responsive release, thereby enhancing the spatiotemporal controllability of antibacterial treatment. For example, materials with micro/nanopores can effectively prevent the aggregation of Ag NPs and improve the sustainability of individual NPs.^[^ ^206^ ^]^ Cu NMs doped in polyurethane, zeolite NPs, aerogels, and hydrogels exhibited enhanced antibacterial properties.^[^ ^207^, ^208^, ^209^, ^210^, ^211^, ^212^ ^]^ Hydrogel 3D networks possess excellent biocompatibility, large pore sizes and volumes, and high specific surface areas.^[^ ^213^, ^214^ ^]^ Stimulus–responsive composite hydrogels can provide mechanical support as well as controlled release for group IB metal‐based NMs.^[^ ^215^ ^]^ For instance, Huang et al. developed pH‐ and temperature‐responsive hydrogels by leveraging the influence of pH on ion interactions and temperature on complexation, allowing stable and prolonged release of Ag, thereby prolonging the effective antibacterial activity (**Figure** **11**A).^[^ ^216^ ^]^ Kong et al. developed hydrogels contained phenylboronic acid‐diol ester bonds with pH‐ and ROS‐responsive drug release under inflammatory stimuli, achieving on‐demand drug administration.^[^ ^217^ ^]^ Du et al. encapsulated Ag_2_S quantum dots in hydrogels that allowed controlled Ag release through volume changes induced by NIR light irradiation, achieving excellent antibacterial performance (Figure 11B).^[^ ^218^ ^]^ Yang et al. by modulation of hydrogen bond strength regulated initial 4 h drug release via weak hydrogen bonds while controlling sustained Cu^2+^ release over 48 h through strong hydrogen bonds, thus achieving optimal antibacterial effects by adjusting dual releases (Figure 11C).^[^ ^219^ ^]^ Pu et al. obtained self‐regulation of a pH‐sensitive hydrogel that accelerated Cu ion release in acidic conditions while slowing down the release at neutral pH value, offering precise treatment with controlled drug release for different phases of wound healing (Figure 11D).^[^ ^220^ ^]^ Wu et al. utilized carboxymethyl chitosan to synthesize 1–3 nm‐diameter Au NPs with minimal self‐aggregation, which effectively captured bacteria with negative surfaces charge for bactericidal activity.^[^ ^221^ ^]^

**Figure 12:**
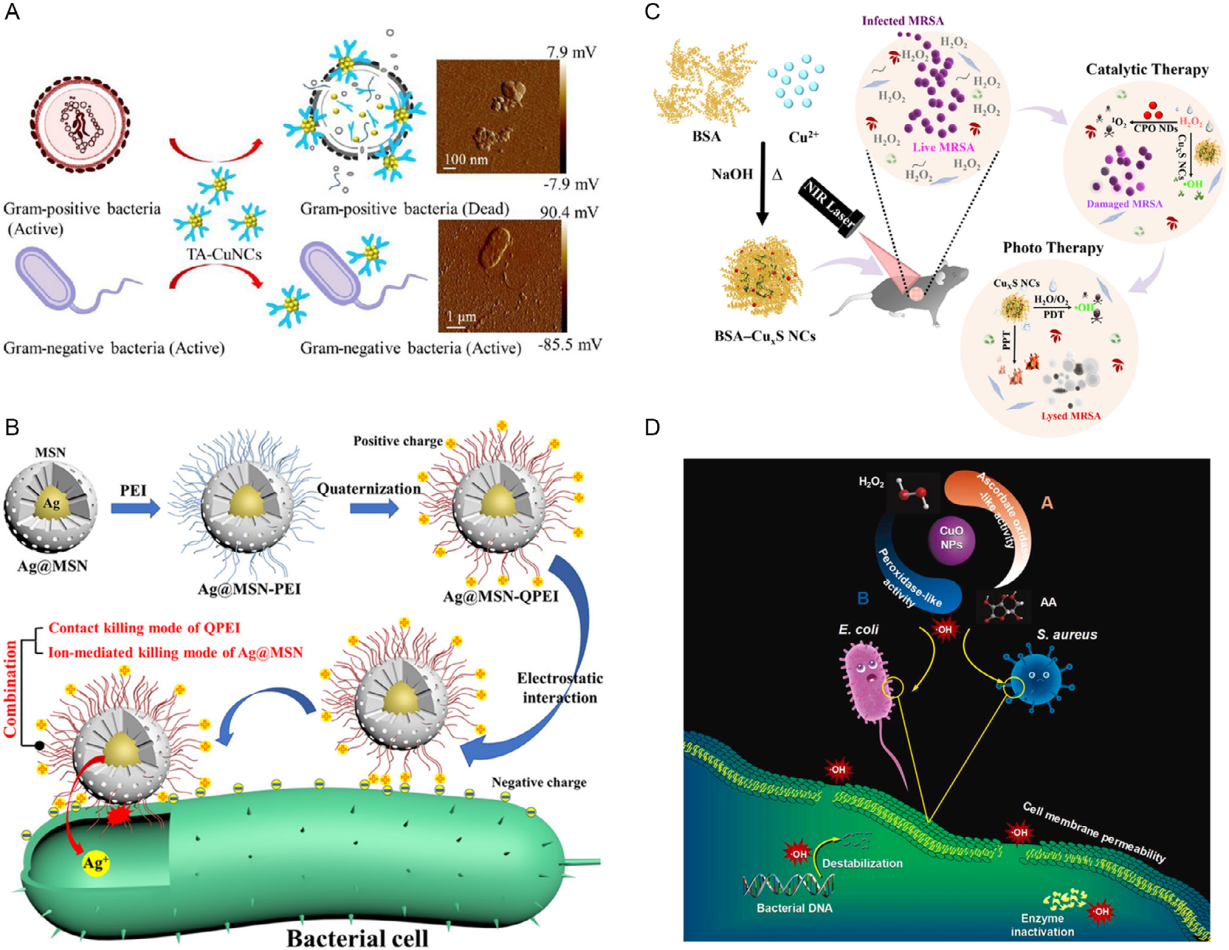
A) Mechanism of selective antibacterial action of TA‐Cu NCs. Reproduced with permission.^[^ ^222^ ^]^ Copyright 2019, Elsevier. B) Formation process and possible interaction of Ag@MSN‐quaternary ammonium polyethyleneimine with bacterial cell. Reproduced with permission.^[^ ^223^ ^]^ Copyright 2021, American Chemical Society. C) Schematic representation of the synthesis of the BSA–Cu_*x*_S NCs and their application in the treatment of bacterial wound infection coupled with NIR laser irradiation. Reproduced with permission.^[^ ^225^ ^]^ Copyright 2021, American Chemical Society. D) Illustration of the possible antimicrobial mechanisms of CuO NP/AA. Reproduced with permission.^[^ ^226^ ^]^ Copyright 2021, Elsevier Ltd.

In addition to serving as drug carriers, organic components can form synergistic antibacterial systems with group IB metal‐based NMs. First, the surface charge of the NMs can be modified by combination with organic components, leading to enhanced targeting effect. Wang et al. utilized tannic acid (TA) to cap Cu NCs, forming TA‐CuNCs, where TA could interact with the peptidoglycan of Gram‐positive bacteria to disrupt their cell membranes.^[^ ^222^ ^]^ The negatively charged TA‐CuNCs could bind to Gram‐positive bacteria through electrostatic interactions, improving the selectivity of TA‐CuNCs for bactericidal action (**Figure** **12**A). The penetration of TA‐CuNCs into the Gram‐positive bacteria may lead to the release of Cu/Cu ions, causing further damage to bacterial cell membrane, inhibition of enzyme activity, alteration in bacterial cell structure, and ultimately inhibition of bacterial growth, demonstrating significant antibacterial effects. Niu et al. modified Ag NPs with the cationic quaternized polyethyleneimine to impart a positive surface charge, achieving targeted interaction with bacterial cell (Figure 12B).^[^ ^223^ ^]^ Second, the antibacterial properties of group IB metal‐based NMs can be directly or indirectly enhanced by the modulation of organic components.^[^ ^224^ ^]^ Chang et al. utilized BSA and 1,3‐propanedithiol to modify Cu NCs.^[^ ^225^ ^]^ The capping of 1,3‐propanedithiol enhanced the antibacterial activity of the protein‐templated Cu NCs, enabling more efficient generation of ROS and consequent disruption of cell membrane integrity (Figure 12C). The MIC of the protein‐templated Cu NCs was at least tenfold lower than that of the BSA‐Cu NCs. Zhuang et al. synthesized CuO NPs/AA by combining ecofriendly AA and CuO NPs, utilizing the AA oxidase‐ and POD‐like activities of CuO NPs for efficient generation of ROS with antibacterial properties (Figure 12D).^[^ ^226^ ^]^ Polydopamine (PDA) NPs possess excellent photostability, long‐term safety, and good biodegradability.^[^ ^227^ ^]^ Upon combination with Ag NPs, the Ag/PDA nanocomposites significantly increased the PTCE by more than two times due to accelerated charge transfer efficiency and enhanced nonradiative transitions of PDA.^[^ ^228^ ^]^ Similarly, the combination of PDA NPs with Cu NPs as well as Au NPs could also lead to enhanced photothermal properties of the nanocomposite, with near‐double PTCE.^[^ ^229^, ^230^ ^]^ Moreover, PDA could bind and fix Ag and Cu ions when used as a coating, slowing ion leaching, enhancing the durability of Cu‐based coatings, and extending their antibacterial activity.^[^ ^231^, ^232^ ^]^ Yuan et al. prepared blue‐emitting Cu NCs (B‐Cu NCs) and cyan‐emitting Cu NCs (C–Cu NCs) with aggregation‐induced emission characteristics by adjusting the aggregation of the surface complexes to regulate the photoluminescence performance of Cu NCs.^[^ ^233^ ^]^ Both B‐Cu NCs and C–Cu NCs showed significant visible light absorption (>600 nm) and the antibacterial efficiency of B‐Cu NCs and C–Cu NCs was around 99% through the synergistic coupling of photodynamic and intrinsic antibacterial effects.

Besides the influence on the intrinsic antibacterial activities, organic components can be used to regulate group IB element‐based NMs to acquire supplementary antibacterial effects.^[^ ^234^ ^]^ Jia et al. chelated Ag NPs with TA and obtained TA‐Ag nanozymes with POD‐like activity due to the harnessed electron transfer between Ag NPs and TA that maintained the dynamic redox equilibrium of phenol quinone, resulting in enhanced catalytic efficiency and notable antibacterial properties.^[^ ^235^ ^]^ Unlike Ag and Cu, Au NPs generally have relatively lower antibacterial properties. More strategies focus on modifying Au NPs with small organic molecules. The combination of the multivalence and permeability of Au NPs and the interaction between modified Au NPs and bacterial surface molecules allowed enhanced antibacterial activity. Le et al. functionalized Au NPs with phenylboronic acid linked through varying ratios of thiol and amine groups, resulting in Au NPs with effective and tunable antibacterial activity.^[^ ^236^ ^]^ Yan et al. introduced thiourea (TU) to Au NCs (Au NC‐Mal), resulting in the formation of [Au(TU)_2_]^+^ and smaller Au NCs.^[^ ^237^ ^]^ This led to increased Au accumulation in bacteria and maintained the oxidation states of Au (Au^I^ and Au^III^), thus exhibiting broad‐spectrum antibacterial activity against multidrug‐resistant bacteria. Amr et al. prepared Au NPs by chemically reducing Au ions with chitosan (CHI) as carrier that reduced aggregation and improved stability of Au NPs.^[^ ^238^ ^]^ Due to the intrinsic antibacterial activity of CHI, a synergistic antibacterial effect was achieved. He et al. developed a self‐assembled carrier‐free antibacterial polyhexamethylene biguanide (PHMB)‐hybridized Au NPs (PHMB@Au NPs).^[^ ^239^ ^]^ PHMB@Au NPs could rapidly aggregate on the surface of *S. aureus*, enabling quick bactericidal action under NIR light irradiation. Currently, it has been discovered that the proportion of surface‐charged ligands can be modulated by anchoring different groups, enabling Au NPs to disrupt bacterial cell walls through a combination of multivalent electrostatic and noncovalent interactions, thereby exhibiting Gram‐selective antibacterial action.^[^ ^240^ ^]^

### 6.4 MOF‐Based Nanocomposites

MOFs are hybrid crystalline structures synthesized by coordinating metal nodes composed of single‐metal ions or small clusters of metal ions, with organic linkers. These structures feature periodic and porous internal architectures, offer high specific surface areas, and uniform pore sizes, and the potential to diverse functionalization, making them highly promising for bioapplications.^[^ ^241^, ^242^ ^]^ MOFs can primarily serve as carriers for group IB metal‐based antibacterial agents, preventing their aggregation and ensuring their uniform distribution. For instance, Zhou et al. utilized an in‐situ immobilization strategy to anchor Ag NPs onto UiO‐66‐NH_2_, enhancing the dispersion and utilization of Ag NPs and thereby reducing their consumption and improving biosafety.^[^ ^243^ ^]^ Guo et al. used highly stable polyMOFs with hierarchical porosity and controlled morphology as carriers for Ag NPs, achieving high Ag loading rates, low MIC values, excellent cell and blood compatibility, and improved wound healing in mice.^[^ ^244^ ^]^ Hu et al. in‐situ reduced ultrasmall Au NPs (UsAuNPs) on ultrathin 2D MOFs, where the interaction between MOFs and Au prevented the aggregation of Au NPs with high surface energy.^[^ ^245^ ^]^ The UsAuNPs/MOFs displayed excellent POD‐like activity due to the synergistic effect of UsAuNPs and 2D MOFs, enabling antibacterial treatment at relatively lower H_2_O_2_ concentrations.

**Figure 13:**
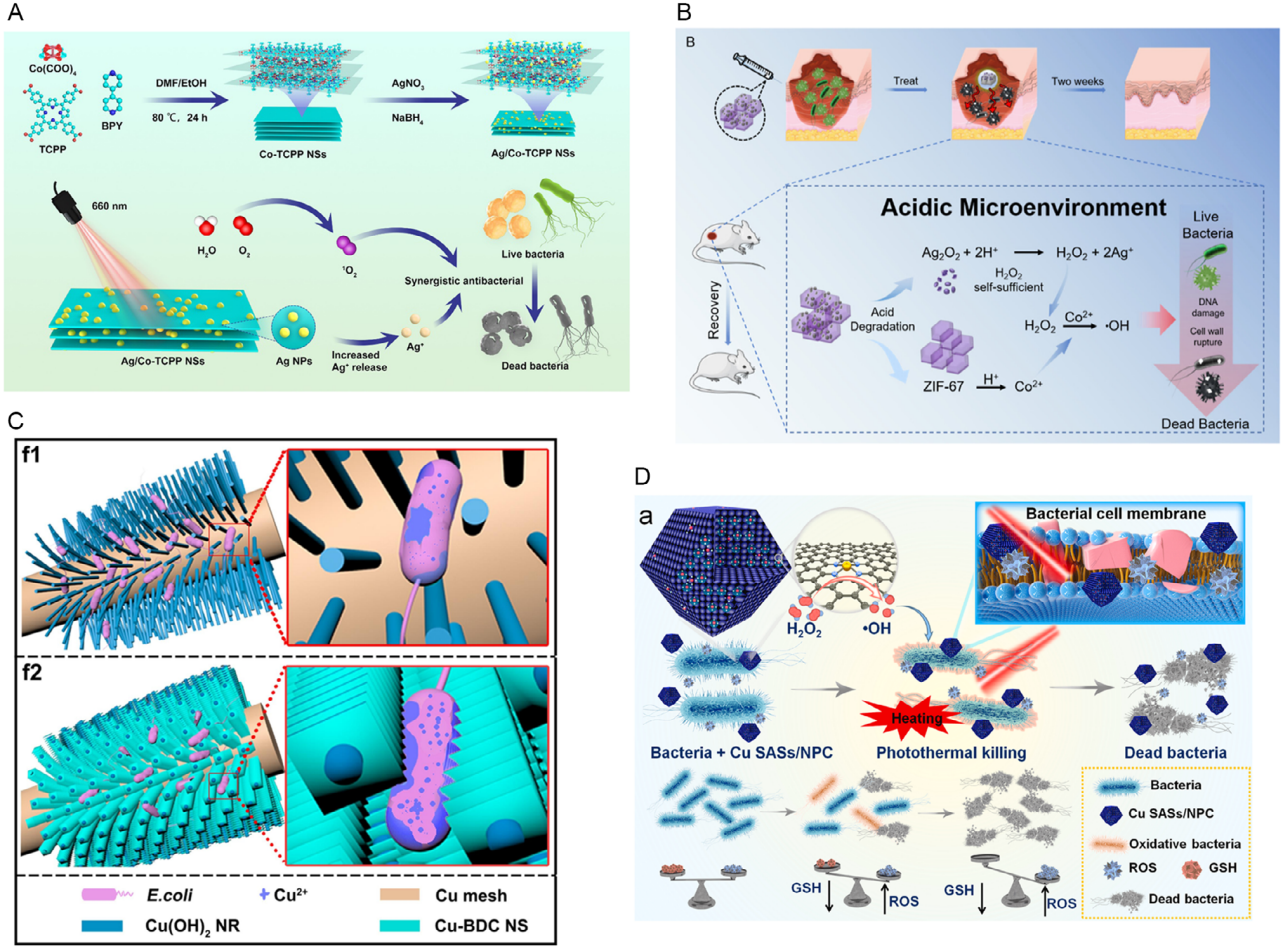
A) Schematic diagram of the PDT synergistic Ag^+^ antibacterial performance of Ag/Co‐TCPP NSs. Reproduced with permission.^[^ ^246^ ^]^ Copyright 2022, Elsevier. B) Illustration of the in vivo antibacterial mechanism of ZIF‐67@Ag_2_O_2_ NSs. Reproduced with permission.^[^ ^247^ ^]^ Copyright 2023, American Chemical Society. C) The antibacterial action of Cu BDC HSs. Reproduced with permission.^[^ ^248^ ^]^ Copyright 2022, Elsevier. D) Antibacterial action of Cu SASs/NPC as GSH‐like mimetic enzyme and HRP‐like nanozyme. Reproduced with permission.^[^ ^250^ ^]^ Copyright 2021, KeAi Publishing Ltd.

MOFs can be ingeniously designed to create novel antibacterial agents with synergistic antibacterial effect, in addition to serving as carriers for group IB metal‐based NMs. Li et al. functionalized 2D MOF Co‐TCPP with Ag NPs to prepare Ag/Co‐TCPP NSs, which exhibited synergistic antibacterial effects.^[^ ^246^ ^]^ Upon irradiation with a 660 nm laser, Ag/Co‐TCPP NSs generated localized ^1^O_2_, creating a highly oxidative environment that promoted partial degradation of Ag NPs, continuously releasing highly toxic Ag^+^, effectively eradicating bacteria by inducing oxidative stress reactions (**Figure** **13**A). Yu et al. developed a pH‐responsive multifunctional ZIF‐67@Ag_2_O_2_ nanoplatform that generated H_2_O_2_ under the mild acidic conditions of the infection microenvironment (Figure 13B).^[^ ^247^ ^]^ Simultaneously, the layered ZIF‐67 NSs could rapidly degrade and release Co^2+^, enhancing antibacterial performance by catalyzing the generated H_2_O_2_. Rosei et al. synthesized a 3D hierarchical structure network (Cu‐BDC HSs) using Cu‐BDC NSs.^[^ ^248^ ^]^ Cu‐BDC HSs combined effects of physical penetration damage induced by sharp NSs and slow, continuous release of Cu ions for chemotherapy, achieving antibacterial efficiency of over 99% after 60 min of treatment (Figure 13C). There was no significant decline in antibacterial activity even after five cycles. Additionally, Cu‐BDC HSs demonstrated excellent structural stability, reusability, and biocompatibility. Liu et al. developed a MOF‐based hybrid nanocatalyst (2D Cu‐TCPP(Fe)/GOx).^[^ ^249^ ^]^ Glucose oxidase (GOx) was employed to catalyze the conversion of glucose into gluconic acid and H_2_O_2_, thereby circumventing the direct utilization of exogenous high‐concentration H_2_O_2_ in tissues. Additionally, the generated gluconic acid effectively lowered the pH value to 3–4, activating the POD‐like activity of the Cu‐TCPP(Fe) NSs and generating •OH for sterilization. Han et al. synthesized Cu_10_MOF by doping PCN‐224 with Cu, utilizing Cu to capture generated electrons and inhibit electron–hole recombination.^[^ ^241^ ^]^ This strategy enhanced the photocatalytic performance and ROS production under 660 nm light irradiation, leading to optimal antibacterial effects through the synergistic action of ROS and heat. Wang et al. synthesized Cu single‐atom sites (SASs)/N‐doped porous carbon nanozyme, where Cu doping significantly enhanced the POD‐ and GPx‐like activities, as well as the photothermal catalytic performance of the nanozyme.^[^ ^250^ ^]^ Upon illumination, heat generation and •OH production occurred, accompanied by GSH consumption, leading to remarkable antibacterial properties (Figure 13D).

## 7 Challenges in the Antibacterial Applications of Group IB Metal‐Based Nanomaterials

With the increasing research and application of metal‐based antibacterial NMs, it has become inevitable that humans and other organisms will be exposed to NPs.^[^ ^251^ ^]^ While acknowledging their remarkable antibacterial properties, concerns about their potential toxicity have also emerged. Most metal‐based NMs including Group IB NMs exhibit broad‐spectrum antibacterial effects through metal ion toxicity, oxidative stress induced by ROS, and photothermal effects, causing damage to normal cells and leading to off‐target toxicity. The extent of this off‐target toxicity varies based on factors such as chemical composition, size, and shape.^[^ ^252^ ^]^ For example, stable metal‐based NMs like Au NPs generally demonstrate better biocompatibility compared to active metal‐based NMs such as Ag NPs and Cu NPs. Smaller NPs possess higher catalytic capacity and toxic potential due to their larger surface area and higher surface atom ratio. Different shapes result in varying crystal surface exposure, biocorona formation, and catalytic capacity, all of which significantly influence cell uptake and cytotoxicity. Additionally, the surface properties of NMs, including surface charge, hydrophobicity, and surface atoms or groups, greatly affect their behavior in both environmental and biological systems. Moreover, the cytotoxicity of NMs may vary depending on the type of cells involved.^[^ ^253^ ^]^

To mitigate off‐target toxicity, targeted delivery is one of the most effective strategies to enhance therapeutic efficacy while minimizing harm to normal cells.^[^ ^254^ ^]^ Drug delivery systems can precisely transport NMs to the intended infection site, significantly reducing toxicity to normal cells. Additionally, for Ag NPs, cytotoxicity is dose dependent, with smaller sizes demonstrating higher toxicity compared to larger ones.^[^ ^255^ ^]^ Spherical Ag NPs exhibit greater toxicity than other shapes. The Ag^+^ ions released from Ag NPs deplete GSH and induce oxidative stress, leading to cell necrosis.^[^ ^256^ ^]^ Furthermore, Ag NPs cause concentration‐dependent DNA damage in various normal cells.^[^ ^257^, ^258^ ^]^ Recent studies have demonstrated that biosynthesized Ag NPs exhibit lower genotoxicity compared to chemically synthesized counterparts.^[^ ^259^ ^]^ Moreover, Ag NPs are less toxic than Ag^+^, while capped silver NPs display the lowest cytotoxicity.^[^ ^260^ ^]^ Notably, the cytotoxicity of Ag NPs increases over time, even at lower exposure levels.^[^ ^261^ ^]^ Unlike Ag, Cu is an essential element in living organisms with trace amounts, playing a critical role in regulating biochemical reactions.^[^ ^262^ ^]^ However, excessive Cu can disrupt cellular Cu homeostasis, elevate ROS levels, induce oxidative stress, cause DNA damage, and trigger apoptosis.^[^ ^263^, ^264^ ^]^ Similar to silver, the most toxic form of copper is the copper ion.^[^ ^265^ ^]^ Therefore, it is crucial to minimize the presence of copper ions. Moreover, studies have shown that Cu NPs within the 40–60 nm range exhibit greater cytotoxicity compared to those with larger or smaller sizes, which may aggregate, thereby reducing cytotoxicity.^[^ ^266^ ^]^ It is important to note that Au NPs are not entirely nontoxic as commonly assumed. Small size or high concentration contributes to increased cytotoxicity of Au NPs, while appropriate modifications can reduce their toxicity.^[^ ^267^, ^268^ ^]^ Positively charged Au NPs can induce DNA damage by creating alkaline unstable sites.^[^ ^269^ ^]^ Combining Au NPs with compounds that mitigate DNA damage, such as lycium barbarum polysaccharides, may effectively reduce this damage.^[^ ^270^ ^]^ Similar to Ag NPs and Cu NPs, Au NPs induce ROS production upon cellular uptake, leading to oxidative stress and cell death, which remains the primary mechanism of toxicity. Additionally, the shape of Au NPs significantly influences their cytotoxicity. For instance, Au nanostars with multibranch surface structures exhibit lower cytotoxicity at concentrations up to 400 μg mL^−1^, whereas spherical Au nanospheres demonstrate higher cytotoxicity.^[^ ^271^ ^]^

In addition to their cytotoxicity, silver and copper are also common environmental pollutants and toxic metals.^[^ ^272^ ^]^ Researchers have examined the impact of Ag NP emissions on freshwater ecosystems in controlled settings. Their findings indicate that while Ag NPs exhibit a low dissolution rate in freshwater, they inhibit diatom growth and cause silver uptake, retention, and long‐term immune stress in gastropods.^[^ ^273^ ^]^ Although Cu NPs are currently used to increase crop yields, if concentrations exceed the physiological tolerance range, the dissolved ions can disrupt cellular homeostasis or induce oxidative stress responses, thereby posing a threat to organisms.^[^ ^274^ ^]^ Au NPs can promote seed germination and seedling growth, yet they may also accumulate in the central nervous system of animals, leading to inflammation in the lungs, liver, and spleen.^[^ ^275^ ^]^ In response to the imminent and existing environmental safety challenges, it is crucial to develop synthesis methods aligned with green chemistry principles, enhance the degradation of NMs in the environment, and reinforce regulatory frameworks for emissions from agriculture and other industries, thereby mitigating the environmental burden posed by these metals.^[^ ^276^ ^]^

In fact, Group IB metal‐based NMs have extensive applications in both the medical field and daily life. They are specifically utilized in medical devices, functional textiles, deodorants, washing machines, kitchenware, and food storage containers.^[^ ^277^ ^]^ Although they have not yet been approved for human consumption, silver‐containing antibacterial dressings have entered clinical trials. For instance, clinical studies have demonstrated that nanosilver dressings can significantly shorten the duration of wound infection treatment cycles.^[^ ^278^ ^]^ Given the wide applications for metal‐based antibacterial NMs, inadequate control can result in issues similar to antibiotic misuse or environmental pollution, ultimately leading to the development of resistance against these metal‐based antibacterial agents. When exposed to sublethal concentrations of NMs, bacteria gradually develop resistance by modifying antioxidant enzyme activity, altering cell membrane permeability, and entering a viable but nonculturable state.^[^ ^279^ ^]^ Metal resistances have developed in bacteria through various mechanisms. Bacteria can reduce their exposure to antibacterial NMs by inducing aggregation of NMs via flagellar proteins or extracellular polymeric substances or by regulating cell membrane composition to decrease permeability or potential.^[^ ^280^, ^281^, ^282^, ^283^ ^]^ Additionally, bacteria can transport NMs out of cells using efflux pumps or active transport mechanisms and repair any damage caused.^[^ ^284^ ^]^ Notably, although bacterial resistance to metal‐based NMs remains stable across multiple generations, it is not heritable because no mutations occur in the coding sequences of the bacterial genome.^[^ ^285^ ^]^ This implies that strategies can be designed to counteract these resistance mechanisms, leading to metal‐based antibiotics with enhanced antimicrobial efficacy. For instance, to address the reduced treatment effectiveness caused by Ag NPs aggregation in *S. aureus* and *E. coli*, approaches such as using pomegranate peel extract to inhibit flagellin and biofilm formation, or immobilizing Ag NPs on stable carriers to prevent aggregation, can be employed.^[^ ^286^ ^]^

## 8 Conclusion and Prospect

Over the past few decades, significant progress has been made in the research of group IB metal‐based NMs in antibacterial applications. NMs with increasingly superior antibacterial performance have been designed for different bacterial strains and specific infection scenarios. The potent broad‐spectrum antibacterial properties make group IB metal‐based NMs a research hotspot in the antibacterial field. In addition to laboratory studies, Ag and Cu have been used as antibacterial coatings on medical implants and medical devices. Ag and Au NPs have been used as active ingredients in creams, soaps, bath gels, masks, and other cosmetics.^[^ ^287^, ^288^, ^289^ ^]^

Despite the above‐mentioned advancements, there is still a considerable gap before their clinical application. Group IB metal‐based NMs, like other metal‐based NMs, are prone to aggregation under complex physiological or environmental conditions, which can result in diminished antibacterial efficacy. Additionally, these materials may exhibit excessive dissolution of metal ions, thereby increasing their toxicity. From the perspective of mechanisms, group IB metal‐based NMs mainly rely on the toxicity of released metal ions and the production of ROS to achieve their antibacterial effects. Unfortunately, both metal ions and ROS can exert significant effects on normal cells and may pose negative impacts on the environment. To address these concerns, future research on Group IB metal‐based antimicrobial NMs should prioritize elucidating their antimicrobial mechanisms, particularly by investigating the detailed molecular pathways involved.

Second, a common and effective strategy is to leverage the interaction between NMs and drug carriers to develop stable composites with superior properties. For instance, utilizing strong metal–carrier can provide advantages such as reduced size, increased payload capacity, and enhanced stability.^[^ ^290^, ^291^ ^]^ This approach minimizes the release and utilization of metal ions and improves therapeutic efficacy while mitigating toxicity to animals, plants, and the environment. Furthermore, carriers with targeting capability, including nanogels, micro/nanomotors, polysaccharides, biomimetic NPs, extracellular vesicles, etc., play a critical role in in vivo application.^[^ ^292^ ^]^ Additionally, the development of intelligent and multifunctional antimicrobial NMs tailored for different application scenarios and needs has always been an important research demand.

The utilization of molecular structures that exhibit high responsiveness and sensitivity to various physical, chemical, or biological stimuli can regulate antibacterial activity through controlled release or activation of antibacterial components. This approach enables intelligent and precise treatment. For instance, MOFs possess flexibility in design, controllable release properties, and excellent synergistic effects, and stability, making them promising candidates for advanced antibacterial applications.^[^ ^293^, ^294^ ^]^ Furthermore, when group IB metal‐based NMs are used in vivo, a part of the material may not be metabolized and excreted. In such cases, reduced residual NMs imply higher biosafety. The investigation into the pharmacokinetics of nanomedicine will contribute significantly to addressing this challenge. Consequently, key research areas for the future may include elucidating the antibacterial mechanisms of materials at the molecular level, developing intelligent antibacterial drugs, studying pharmacokinetics in depth, and achieving more precise targeted delivery.

Finally, a stringent approval process is essential to establish the safety and efficacy of nanomedicine for clinical or large‐scale application. This process, encompassing preclinical studies and clinical trials, needs to be managed by authoritative and professional regulatory bodies such as the FDA, European Medicines Agency, and China National Medical Products Administration to ensure that both the procedural integrity and the final products meet stringent standards. Currently, the regulation of nanotechnology remains inconsistent and ambiguous, with varying guidelines across different regions.^[^ ^295^ ^]^ Therefore, we advocate for a case‐by‐case approach to the safety and risk assessment of NMs. In addition to safety concerns, several critical issues in the application of NMs, including industrial‐scale preparation and production, long‐term stability and shelf life, and economic viability, must also be addressed. Among them the industrial‐scale preparation of NMs remains one of the ultimate challenges, necessitating precise control over NP growth through manipulation of process parameters.^[^ ^296^ ^]^ In practice, certain operating parameters may have minimal direct impact on NM properties but can strongly interact with other parameters, such as temperature, homogenization pressure, and cycle number. Therefore, meticulous optimization of each synthesis parameter is needed. The final Standard Operating Procedures (SOPs should include a Certificate of Analysis (COA), Material Safety Data Sheets (MSDS), and Technical Data Sheets (TDS) to establish a robust foundation for industrial production.^[^ ^297^ ^]^ The complexity of NM design and synthesis also introduces cost‐related challenges. A multistep production process involving various methods can significantly increase production costs.^[^ ^298^ ^]^ Hence, simplifying industrial production processes to achieve cost‐effectiveness comparable to that of clinically used general‐purpose drugs is also of great importance.

In conclusion, group IB metal‐based NMs have been extensively and deeply studied for antimicrobial. Although several challenges remain to be addressed before their potential replacement of antibiotics as a “next‐generation of antibiotic”, there is substantial promise for their antibacterial application. This review aims to offer valuable insights and inspiration for ongoing research on optimization of the antimicrobial performance and biosafety of these NMs, thereby enabling their future application in medical fields.

## Conflict of Interest

The authors declare no conflict of interest.

## Author Contributions

**Xuezhi Zhao**: conceptualization (lead); writing—original draft (lead); writing—review & editing (lead). **Hongyu Wang**: conceptualization (lead); writing—original draft (supporting); writing—review & editing (equal). **Yun Sun**: writing—review & editing (supporting). **Jin Zhang**: conceptualization (equal); project administration (supporting); writing—review & editing (equal). **Huiyu Liu**: funding acquisition (lead); project administration (lead); supervision (lead); writing—review & editing (lead). **Xuezhi Zhao** and **Hongyu Wang** contributed equally to this work.
